# The AR/miR-185-5p/TGF-β axis in renal tubular epithelial cells may regulate hyperuricemia-induced renal crystal deposition by impairing macrophage recruitment and M2 polarization

**DOI:** 10.1371/journal.pone.0360746

**Published:** 2026-11-11

**Authors:** Yulin Yuan, Linkang Yu, Chuanjie Li, Yining Li, Xinyi Lei, Cong Li

**Affiliations:** 1 Department of Urology, Tongji Hospital, Tongji Medical College, Huazhong University of Science and Technology, Wuhan, Hubei, China; 2 Medical College, Wuhan University of Science and Technology, Wuhan, Hubei, China; 3 Hubei Province Key Laboratory of Occupational Hazard Identification and Control, School of Public Health, Wuhan University of Science and Technology, Wuhan, Hubei, China; 4 Hubei Provincial Clinical Research Center for Minimally Invasive Treatment of Urology, Wuhan, Hubei, China; China Medical University, TAIWAN

## Abstract

This study investigated whether androgen receptor (AR) signaling in renal tubular epithelial cells contributes to altered macrophage responses and renal crystal deposition under hyperuricemic conditions. We integrated clinical correlation analyses, in vitro genetic manipulations in HK-2 cells and THP-1 macrophage co-cultures, and an in vivo rat model of hyperuricemia-induced crystalluria. Uric acid exposure increased AR and miR-185-5p expression in HK-2 cells. In co-culture experiments, elevated miR-185-5p suppressed TGF-β secretion, altered macrophage migration, increased M1-associated markers, and decreased M2-associated markers. In vivo, targeted inhibition of AR or miR-185-5p significantly restored renal TGF-β levels, increased M2-associated marker expression, attenuated tubular injury, and markedly reduced crystal deposition compared to untreated hyperuricemic controls. Furthermore, pharmacological blockade using enzalutamide mirrored these protective effects. In conclusion, we identify a novel pathogenic cascade where uric acid-driven AR signaling disrupts the renal immune microenvironment through miR-185-5p-mediated TGF-β suppression, ultimately driving renal crystal deposition.

## Introduction

Nephrolithiasis is a prevalent and recurrent urological disorder associated with significant morbidity, including severe pain, renal damage, and an increased risk of chronic kidney disease [1,2]. The pathogenesis of kidney stones is multifactorial, involving complex interactions between metabolic disturbances, urinary supersaturation, and local tissue responses [3,4]. Among the established metabolic risk factors, hyperuricemia is particularly prominent, being closely linked to the development and progression of urolithiasis [5,6]. Elevated serum uric acid (UA) levels contribute to a pro-inflammatory and oxidative environment within the kidney, which can promote crystal nucleation, growth, and retention [7].

Notably, our recent large-scale cross-sectional study in the Chinese population revealed a striking sex disparity in this association: while serum UA levels are positively correlated with the risk of kidney stone formation in a dose-response manner in men, this association is notably absent in women [8]. This finding suggests that sex-specific factors, rather than UA alone, play a decisive role in the pathophysiology of stone formation. Given that testosterone is a known predisposing factor for kidney disease and has been implicated in promoting crystal-cell adhesion in renal tubular epithelial cells (RTECs) [9–11], we hypothesized that the androgen receptor (AR) might serve as a critical molecular bridge linking hyperuricemia to the observed male-predominant stone risk.

Current research has established that the local renal immune microenvironment, particularly macrophage behavior, plays a pivotal role in determining the fate of deposited crystals [12–14]. Macrophages, which can polarize into pro-inflammatory (M1) or anti-inflammatory/pro-fibrotic (M2) phenotypes, are essential for phagocytosing crystals and modulating tissue repair [13]. In the context of crystal nephropathy, a shift towards the M2 phenotype is generally associated with enhanced crystal clearance and tissue resolution [12]. Furthermore, RTECs, as the primary interface with filtered uric acid and crystals, are not passive bystanders but active participants in this process, potentially secreting signals that orchestrate immune cell recruitment and function [15,16]. While the cytokine transforming growth factor-beta (TGF-β) is a known regulator of macrophage polarization and fibrotic responses, and microRNAs (miRNAs) have emerged as key post-transcriptional modulators in renal diseases, the specific signaling cascade initiated by uric acid in RTECs that converges on macrophage dysfunction remains largely unexplored [17–19].

This knowledge gap is significant because a detailed mechanistic understanding is a prerequisite for moving beyond symptomatic management to mechanism-based prevention. Specifically, it is unknown whether and how uric acid stimulation of RTECs regulates a defined signaling axis involving AR, specific miRNAs like miR-185-5p, and downstream effectors such as TGF-β to alter macrophage phenotype and phagocytic capacity. Addressing this gap is necessary to identify novel, druggable targets within the pathogenic pathway from hyperuricemia to stone formation.

To bridge this gap, the present study employs an integrative translational strategy—spanning clinical correlation, in vitro mechanistic dissection, and in vivo validation—to systematically investigate the role of the AR/miR-185-5p/TGF-β axis in hyperuricemia-induced nephrolithiasis. By combining our previous clinical insights with sophisticated cellular models, including genetic manipulation of RTECs and Transwell co-culture systems, we aim to: (1) correlate clinical indices of this pathway with macrophage polarization and stone severity; (2) mechanistically dissect the regulation of the AR/miR-185-5p/TGF-β cascade in UA-stimulated RTECs; (3) characterize the paracrine effects of this epithelial signaling on macrophage migration, polarization, and crystal phagocytic capacity; and (4) evaluate whether pharmacological or genetic modulation of this axis can attenuate macrophage dysfunction and renal crystal deposition in a rat model of hyperuricemic crystalluria. Ultimately, this comprehensive approach seeks to establish a causal link between epithelial metabolic stress and immune-mediated crystal clearance, thereby identifying novel therapeutic targets for the prevention of recurrent urolithiasis.

## Materials and methods

### 1. Cell culture and treatment

Human renal tubular epithelial cells (HK-2) and human monocytic leukemia cells (THP-1) were purchased from the American Type Culture Collection (ATCC). HK-2 cells were cultured in DMEM/F12 medium supplemented with 10% fetal bovine serum (FBS). THP-1 cells were differentiated into macrophages using 100 ng/mL phorbol 12-myristate 13-acetate (PMA) for 72 h. To simulate hyperuricemia (HUC), HK-2 cells were treated with uric acid (20 mg/dL or 50 mg/dL) for 24 h.

### 2. Quantitative Reverse Transcription (q)PCR

Total RNA was extracted using TRIzol reagent. cDNA was synthesized and amplified using SYBR Green Master Mix. Relative expression levels were calculated using the 2 ⁻ ΔΔCt method, with GAPDH as an internal control. qPCR was performed in accordance with standard techniques as reported previously [20].

### 3. Western blotting

Total protein was extracted using RIPA buffer. Proteins were separated by SDS-PAGE, transferred to PVDF membranes, and incubated with primary antibodies against AR (22089–1-AP, Proteintech), GAPDH (60004–1-Ig, Proteintech), ACTIN (66009–1-Ig, Proteintech), iNOS (22226–1-AP, Proteintech) and ARG1 (16001–1-AP, Proteintech). Western blotting assays were performed in accordance with standard techniques, as reported previously [21].

### 4. ELISA

TGF-β levels in the cell culture supernatants were measured using commercial ELISA kits according to the manufacturer’s protocols.

### 5. Genetic manipulation

Stable AR-knockdown (shAR) and AR-overexpression (OE-AR) HK-2 cell lines were constructed using CRISPR/Cas9 or lentiviral vectors. The corresponding negative-control groups for AR knockdown and AR overexpression were designated shNC and OE-NC, respectively. Similarly, mimics and inhibitors were transfected into HK-2 cells using Lipofectamine 3000 (Invitrogen) according to the manufacturer’s instructions. Transfection efficiency was validated by qPCR and western blotting.

### 6. Co-culture system and macrophage functional assays

A Transwell co-culture system (0.4 μm pore size) was employed to investigate the crosstalk between HK-2 cells and macrophages. HK-2 cells were seeded in the lower chamber, and PMA-differentiated THP-1 macrophages were seeded in the upper chamber.

### 7. Migration assay

Macrophage migration was assessed using a Transwell chamber (5.0 μm pore size). After 20 h, non-migrated cells were removed, and migrated cells were fixed with methanol, stained with crystal violet, and counted under a microscope.

### 8. Phagocytosis assay

Macrophages were incubated with Ponceau S-labeled calcium oxalate crystals for 24 h. The percentage of macrophages containing labeled crystals was analyzed via optical microscopy.

### 9. Flow cytometry

Macrophage polarization was identified using fluorochrome-conjugated antibodies (PE-CD86, APC-ARG1, BV605-CD206, BV421-CD68) and analyzed on a flow cytometer.

### 10. Animal models

Male Sprague-Dawley (SD) rats (8 weeks old) were used for in vivo studies. Hyperuricemia was induced by oral administration of yeast extract (10−15 g/kg/d) combined with intraperitoneal injection of potassium oxonate (250−300 mg/kg/d). To investigate the role of the AR/miR-185-5p axis, rats were subjected to renal subcapsular injection of AAV-sh-AR or AAV-sh-miR-185-5p. Renal crystal deposition was induced by glyoxylate (80 mg/kg/d) for 6 days.

### 11. Histological and pathological evaluation

Renal tissues were harvested and fixed in 4% paraformaldehyde. Von Kossa staining was used to detect calcium crystal deposition. PAS staining was used to assess renal histopathological changes, whereas TUNEL staining was used to detect DNA fragmentation. Co-localization of ARG1 and iNOS was performed to visualize macrophage distribution and polarization status in renal tissues using immunofluorescence.

### 12. Statistical analysis

Data are presented as mean ± standard deviation (SD). Statistical significance between groups was determined using Student’s t-test or one-way ANOVA followed by Tukey’s post-hoc test. A *P* value < 0.05 was considered statistically significant. All analyses were performed using GraphPad Prism 9.0 software.

### 13. Ethics statement

All animal procedures were approved by the IACUC of Wuhan University of Science and Technology Medical Ethics Committee (No.2026162), following ARRIVE guidelines.

### 14. Euthanasia

Drug injection: Rats that meet the humane endpoints will be euthanized with sodium pentobarbital or an overdose of ketamine-diazepam mixture. This procedure will be performed by experimental personnel trained by the Animal Welfare Committee. Adjuvant anesthesia method: After general anesthesia, euthanasia can be completed by cervical dislocation or overdose of anesthetic drugs to ensure painless and rapid death.

### 15. Specific euthanasia criteria for experimental rats

1) Body weight loss exceeding 20%: If a rat loses more than 20% of its body weight during the experiment, it will be regarded as reaching the humane endpoint. The experiment will be terminated immediately and the rat will be humanely euthanized.2) Obvious behavioral changes or physical discomfort: If a rat shows persistent hypoactivity, tachypnea, abnormal behaviors (such as persistent licking, moaning, convulsions, etc.), unresponsiveness, or acute pain (such as persistent vomiting, convulsions, etc.), the experiment will be terminated immediately and the rat will be humanely euthanized.3) Abnormal physiological indicators: If a rat develops tachypnea, obvious dehydration, inability to eat or drink, or severe symptoms such as abdominal distension, liver and kidney injury, the experiment will be terminated immediately.4) End of the study period: After the experiment is completed, all rats will be euthanized at the preset endpoint (day 22) to avoid unnecessary suffering.

## Results

### 1. Uric acid upregulates AR expression in HK-2 cells

To investigate the role of androgen receptor (AR) in hyperuricemia-induced renal crystal deposition, we first examined AR expression in human renal tubular epithelial cells (HK-2). qPCR and western blotting results demonstrated that high-concentration uric acid (20 mg/dL and 50 mg/dL) treatment significantly increased both the mRNA and protein levels of AR in HK-2 cells in a dose-dependent manner (Fig 1A–1B). To further explore the function of AR, we successfully constructed AR-knockdown (shAR) and AR-overexpression (OE-AR) HK-2 cell lines, with the efficiency verified by qPCR and western blotting (Fig 1C–1D).

**Fig 1:**
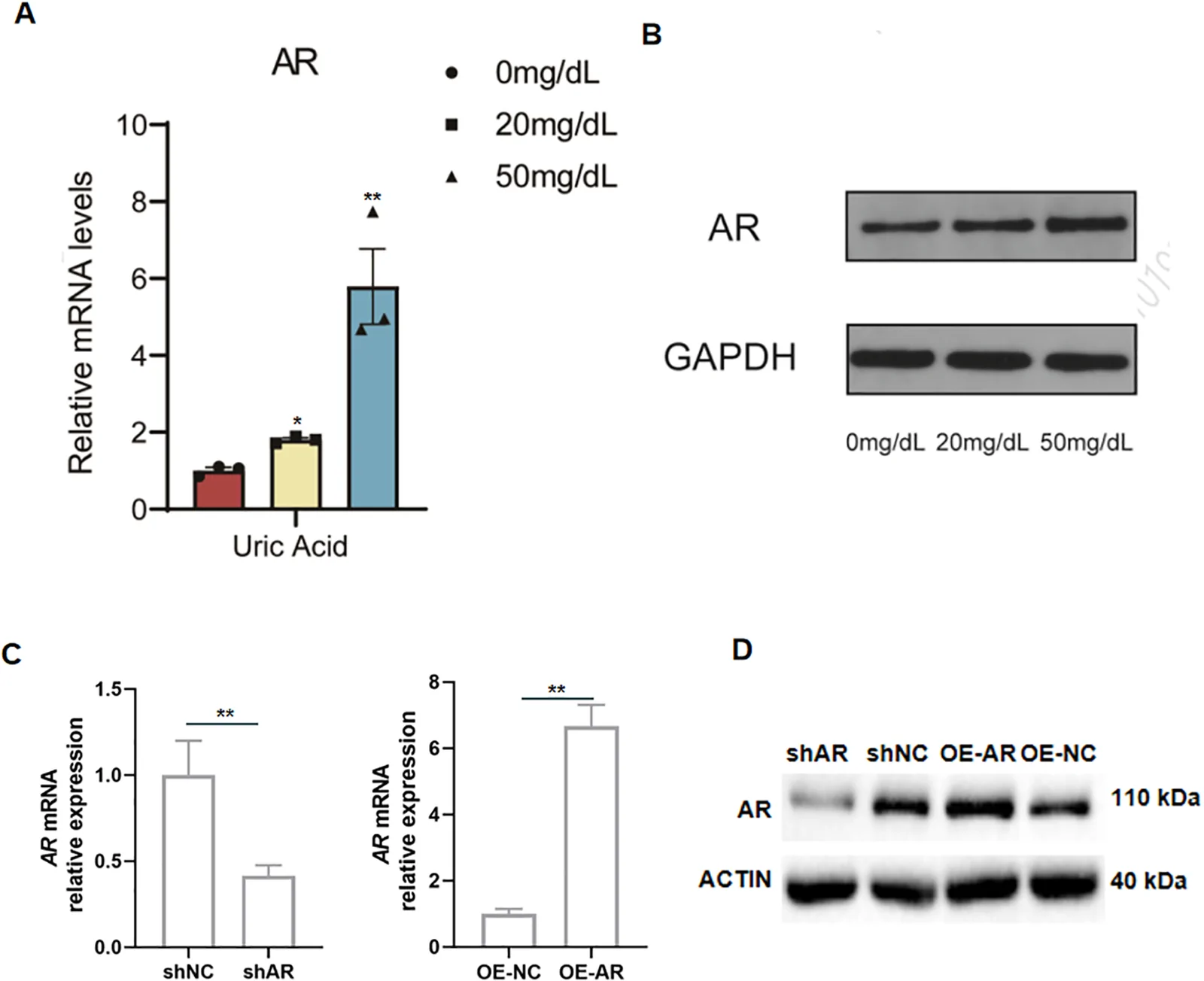
Uric acid enhances AR expression. (A-B) AR expression in HK-2 cells treated with uric acid was detected by qPCR and western blotting. (C-D) The efficiency of AR knockdown and overexpression was verified by qPCR and western blotting. **, *P* < 0.01.

### 2. AR modulation affects macrophage M1- and M2-associated markers under high-uric-acid conditions

Having verified that uric acid upregulates AR expression in HK‑2 cells, we then investigated whether epithelial‑derived AR activity affects macrophage migration as well as M1/M2 polarization. Transwell migration assays showed reduced migration of THP-1-derived macrophages toward supernatants from uric acid-treated HK-2 cells compared with control supernatants (Fig 2C). Western blotting and flow cytometric analyses revealed that uric acid treatment was associated with increased expression of M1-associated markers (including iNOS and CD86) and decreased expression of M2-associated markers (including CD163 and CD206) in macrophages. Notably, AR knockdown reversed these effects, whereas AR overexpression exacerbated the M1 polarization induced by uric acid (Fig 2A, 2D). Consistent with these findings, qPCR analysis confirmed that uric acid treatment decreased the expression of M2 markers (CD163, CD206) and increased M1 markers (iNOS, TNF-α, IL-1β) (Fig 3A, 3C–3F). Because TGF-β is a key cytokine implicated in M2 polarization, we next assessed whether AR modulation was associated with changes in TGF-β levels. Uric acid treatment was associated with lower TGF-β levels, and AR knockdown partially attenuated this decrease (Fig 2B; Fig 3B).

**Fig 2:**
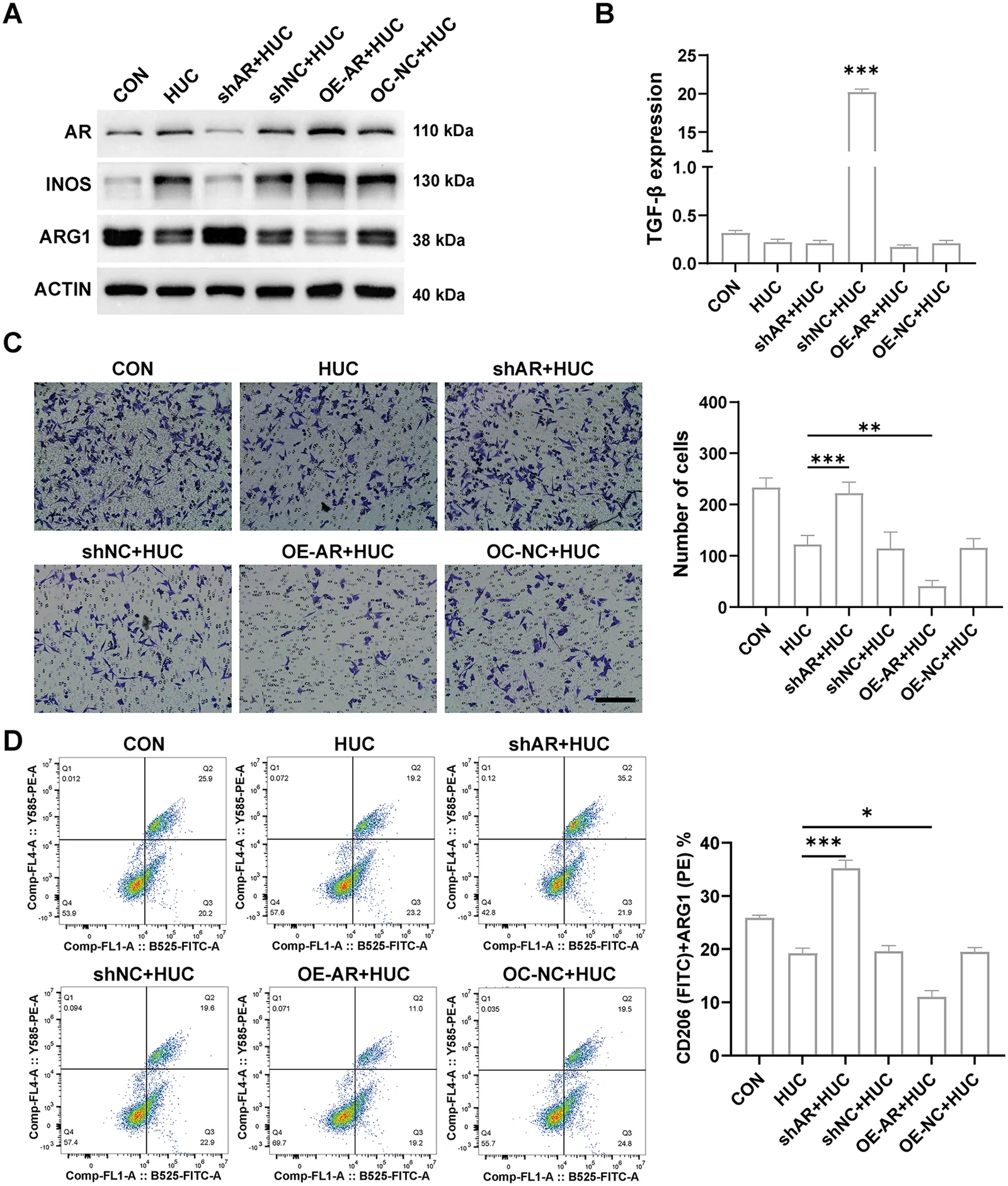
Hyperuricemia may contribute to changes in macrophage M1- and M2-associated markers by upregulating AR. (A) Western blotting analysis of the effects of uric acid levels and AR expression on macrophage polarization markers. (B) qPCR analysis of the effects of uric acid levels and AR expression on TGF-β expression. (C) Transwell assay assessing the effects of uric acid levels and AR expression on macrophage migration. Scale bar, 150 μm. (D) Flow cytometric analysis of the effects of uric acid levels and AR expression on macrophage polarization. *, *P* < 0.05; ***, *P* < 0.001.

**Fig 3:**
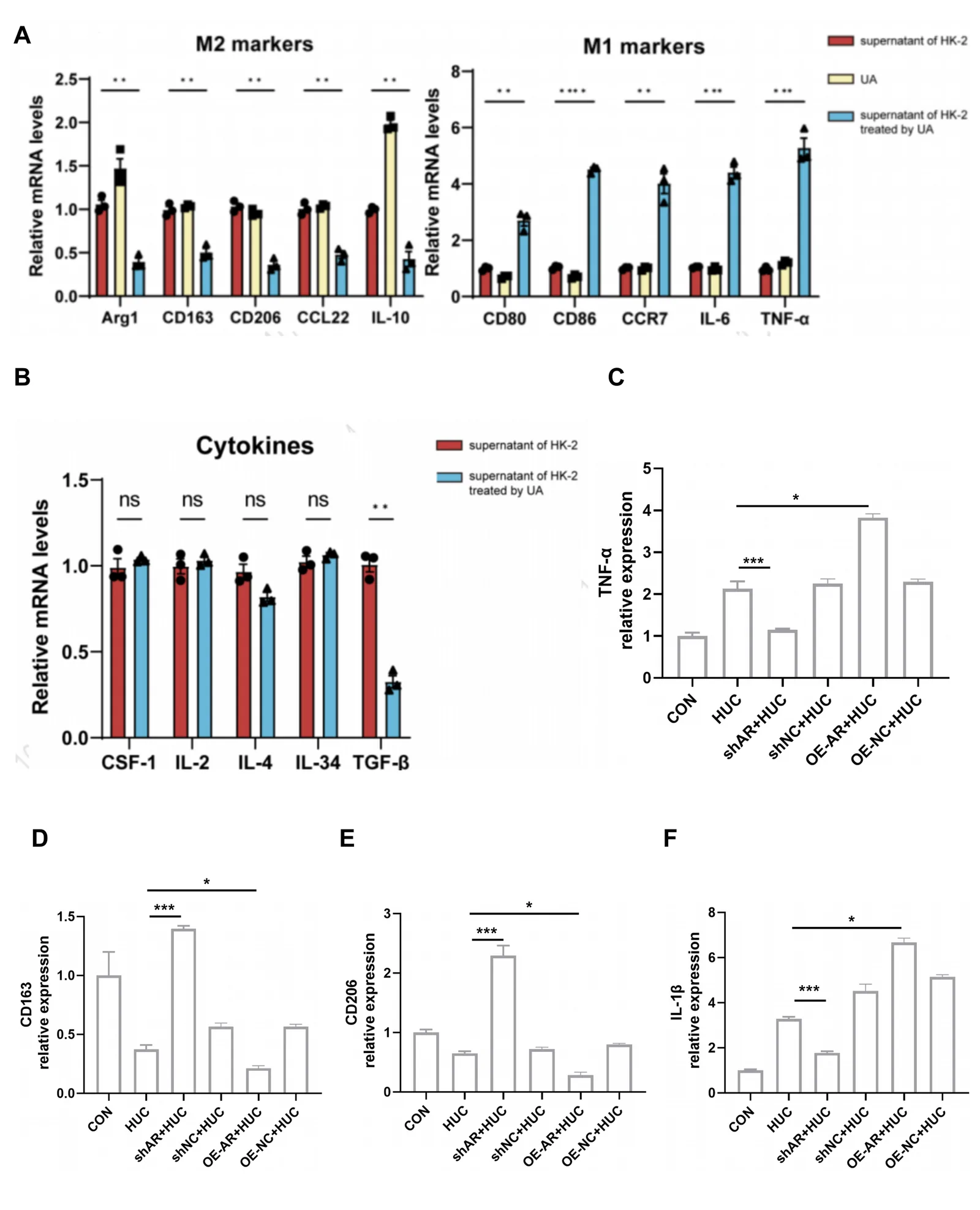
Hyperuricemia influences inflammatory cytokine secretion by upregulating AR. (A) qPCR analysis of the effects of uric acid levels on M2 and M1 macrophage marker expression. (B) qPCR analysis of the effects of uric acid levels on the expression of CSF-1, IL-2, IL-4, IL-34, and TGF-β. (C) qPCR analysis of the effects of uric acid levels and AR expression on TNF-α expression. (D) qPCR analysis of the effects of uric acid levels and AR expression on CD163 expression. (E) qPCR analysis of the effects of uric acid levels and AR expression on CD206 expression. (F) qPCR analysis of the effects of uric acid levels and AR expression on IL-1β expression. *, *P* < 0.05; ***, *P* < 0.001.

### 3. miR-185-5p acts as a potential target of AR in regulating TGF-β

Because AR-dependent changes in TGF-β accompanied altered macrophage responses, we next used a bioinformatic intersection analysis to identify miR-185-5p as a potential target of AR-dependent TGF-β regulation (Fig 4A). qPCR validation confirmed that miR-185-5p expression was significantly upregulated in HK-2 cells under high uric acid conditions (Fig 4C; Fig 7A). Functional assays demonstrated that miR-185-5p mimics suppressed TGF-β expression and promoted M1 macrophage polarization, while miR-185-5p inhibitors exerted the opposite effects (Fig 4D–4H, 7B–7E). Furthermore, co-culture experiments confirmed that miR-185-5p modulation significantly altered macrophage migration and polarization profiles (Fig 5A–5B).

**Fig 4:**
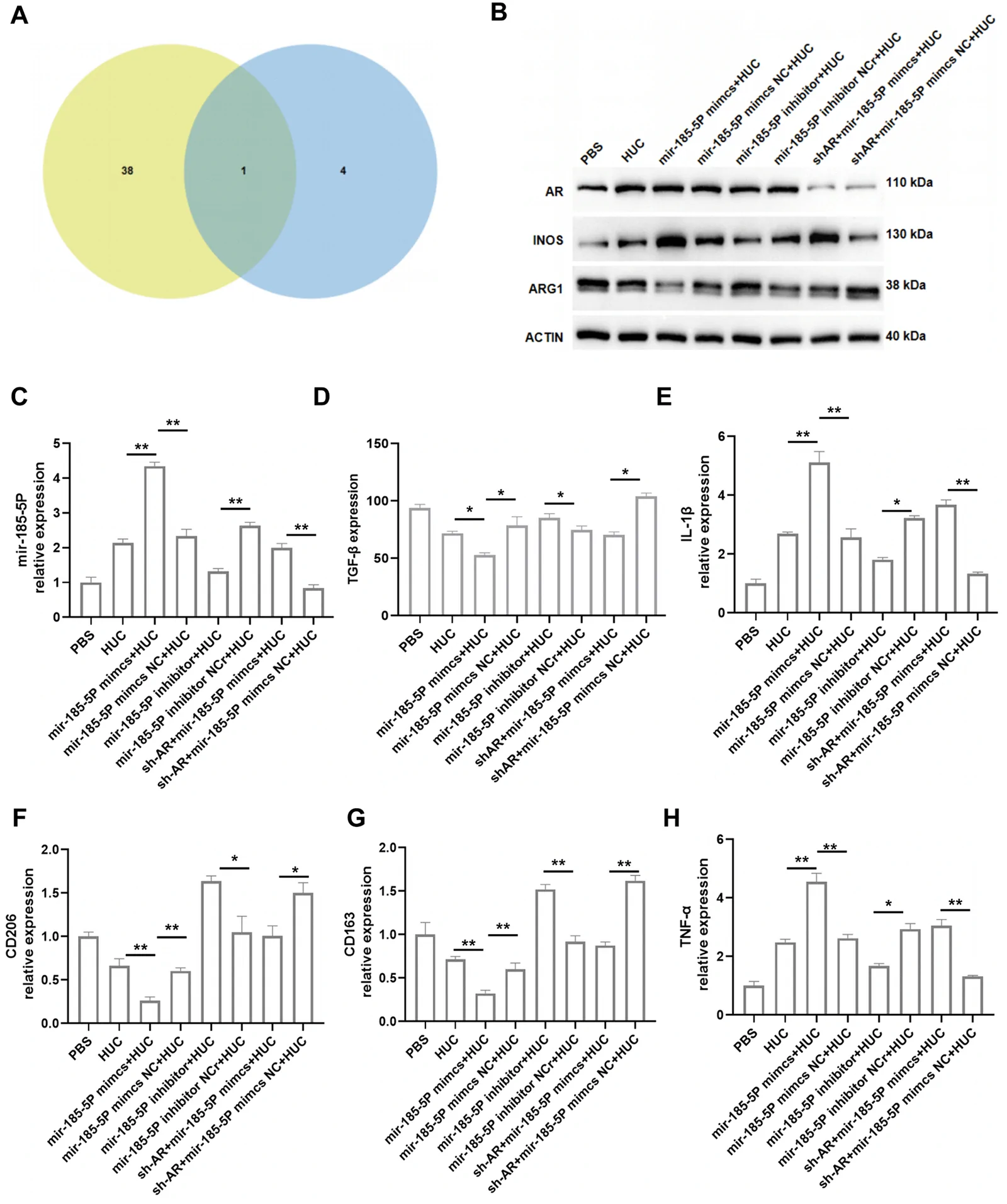
miR-185-5p is a key factor in AR-mediated regulation of TGF-β expression. (A) Venn diagram screening identifying miR-185-5p. (B) Western blotting analysis of the effects of uric acid levels and miR-185-5p expression on macrophage polarization markers. (C) qPCR validation of miR-185-5p expression. (D) qPCR analysis of the effects of uric acid levels and miR-185-5p expression on TGF-β expression. (E) qPCR analysis of the effects of uric acid levels and miR-185-5p expression on IL-1β expression. (F) qPCR analysis of the effects of uric acid levels and miR-185-5p expression on CD206 expression. (G) qPCR analysis of the effects of uric acid levels and miR-185-5p expression on CD163 expression. (H) qPCR analysis of the effects of uric acid levels and miR-185-5p expression on TNF-α expression. *, *P* < 0.05; **, *P* < 0.01; ***, *P* < 0.001.

**Fig 5:**
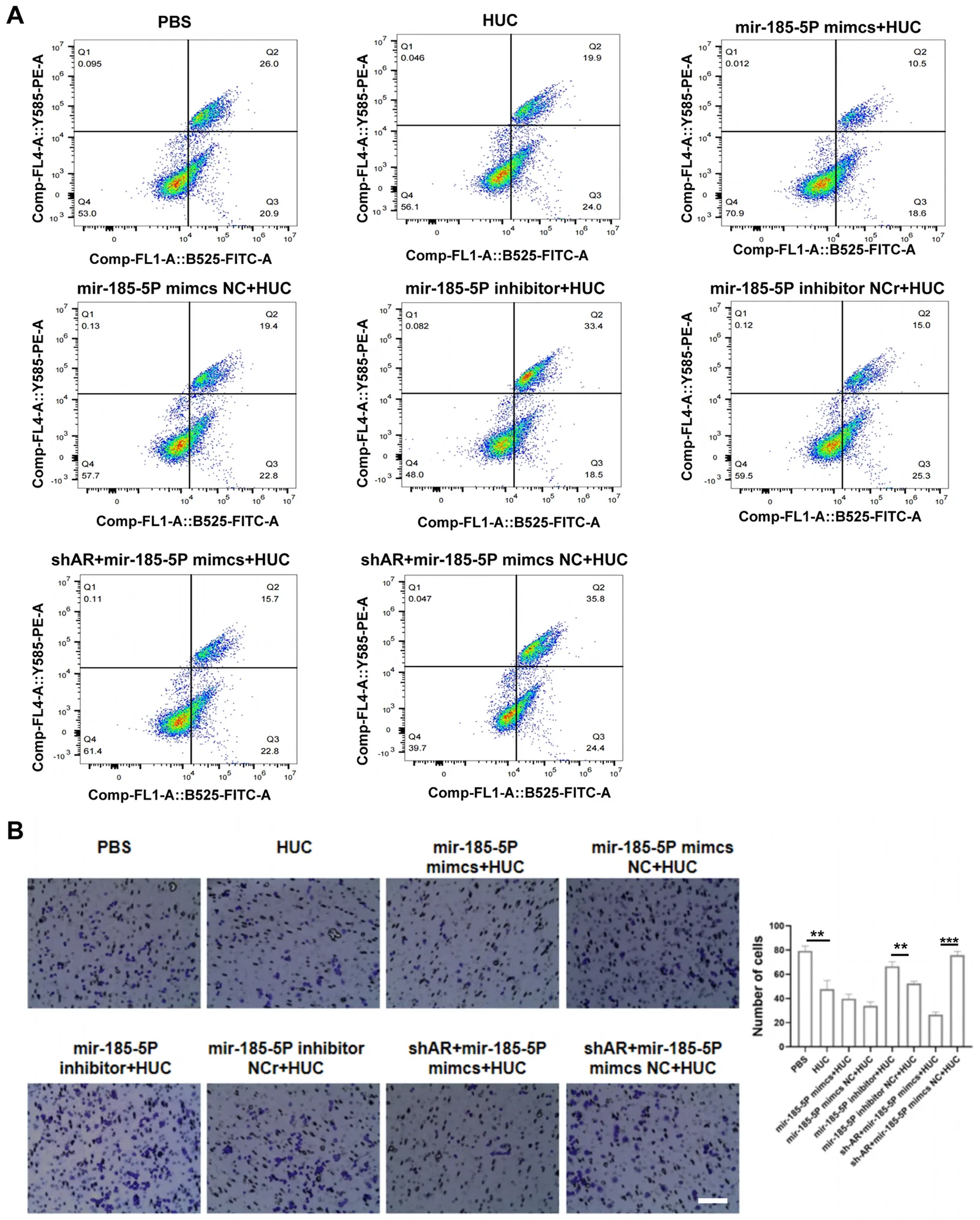
miR-185-5p is a key factor in AR-mediated regulation of TGF-β expression. (A) Flow cytometric analysis of the effects of uric acid levels and miR-185-5p expression on macrophage polarization. (B) Transwell assay assessing the effects of uric acid levels and miR-185-5p expression on macrophage migration. Scale bar, 150 μm. **, *P* < 0.01.

### 4. Targeted inhibition of AR or miR-185-5p may suppress renal crystal deposition in vivo

Having identified an AR/miR-185-5p/TGF-β pathway associated with macrophage responses in vitro, we next tested whether pharmacological or genetic inhibition of this axis reduced renal crystal deposition in vivo. Von Kossa staining showed that enzalutamide treatment or miR-185-5p knockdown significantly reduced calcium crystal deposition in the renal papillae of hyperuricemic rats (Fig 6A). Additionally, TUNEL staining showed fewer TUNEL-positive cells following enzalutamide treatment or miR-185-5p knockdown (Fig 6B-6C). To further elucidate the molecular mechanism, we examined whether miR-185-5p is involved in AR-regulated TGF-β expression. qPCR validation confirmed that miR-185-5p expression was modulated as expected (Fig 7A). Notably, enzalutamide treatment or miR-185-5p knockdown significantly reduced the expression of pro-inflammatory factors TNF-α and IL-1β (Fig 7B–7C), while increasing the expression of M2 macrophage markers CD206 and CD163 (Fig 7D–7E). These results suggested that miR-185-5p acts as a key downstream effector of AR in regulating TGF-β expression and inflammatory responses. Collectively, the AR/miR-185-5p/TGF-β axis plays a critical role in the pathogenesis of hyperuricemia-associated nephrolithiasis, and targeting this pathway may represent a promising therapeutic strategy.

**Fig 6:**
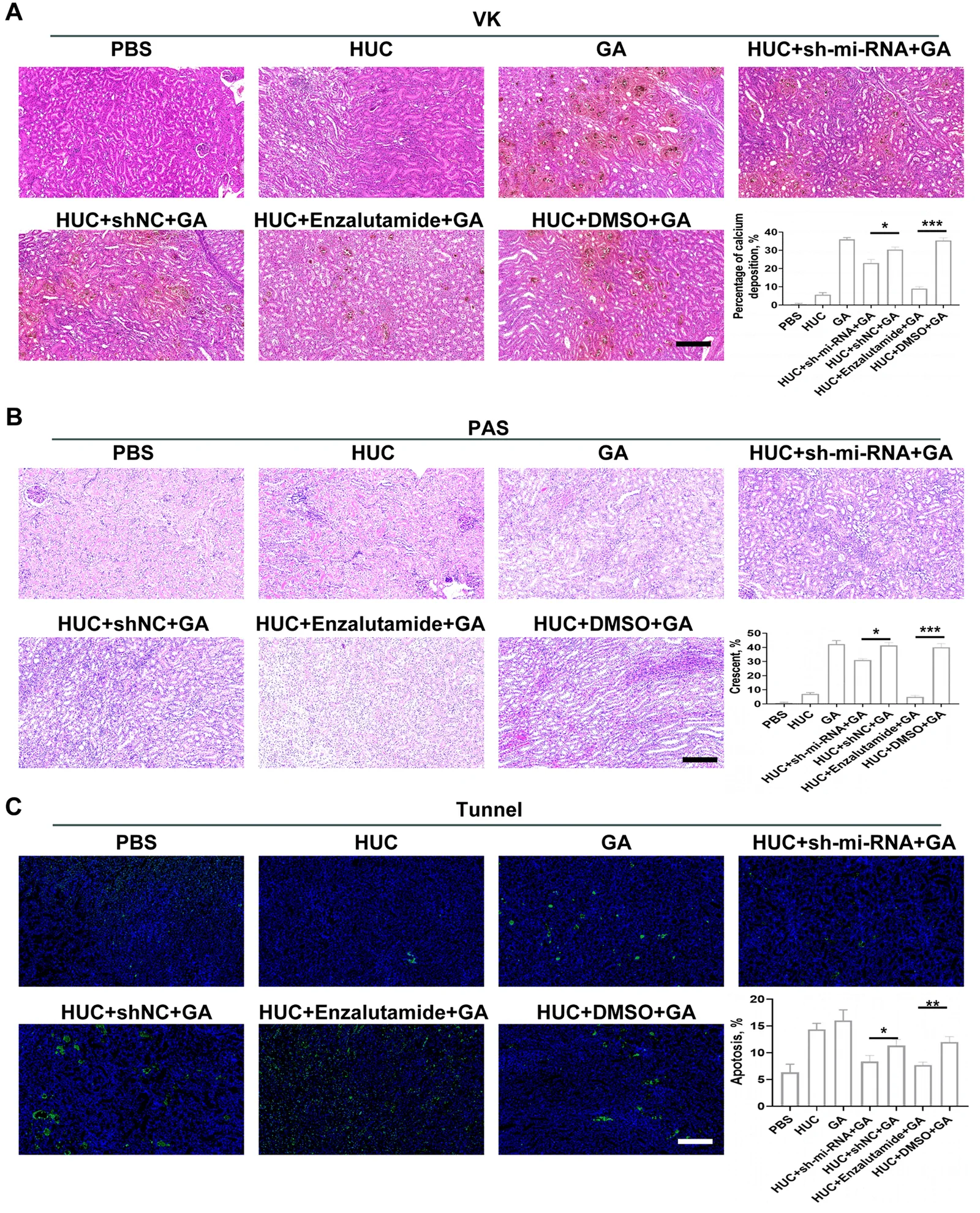
Targeted inhibition of AR or miR-185-5p effectively suppresses renal crystal deposition. (A) Von Kossa staining was performed to verify whether enzalutamide treatment or knockdown of miR-185-5p inhibits crystal deposition in renal papillary tissue. Scale bar, 300 μm. (B) PAS staining was used to evaluate the effects of enzalutamide treatment or miR-185-5p knockdown on renal tubular epithelial cell injury and necrosis in kidney tissue. Scale bar, 300 μm. (C) TUNEL staining was performed to assess DNA fragmentation in renal tissue following enzalutamide treatment or miR-185-5p knockdown. Scale bar, 300 μm. *, *P* < 0.05; ***, *P* < 0.01..

**Fig 7:**
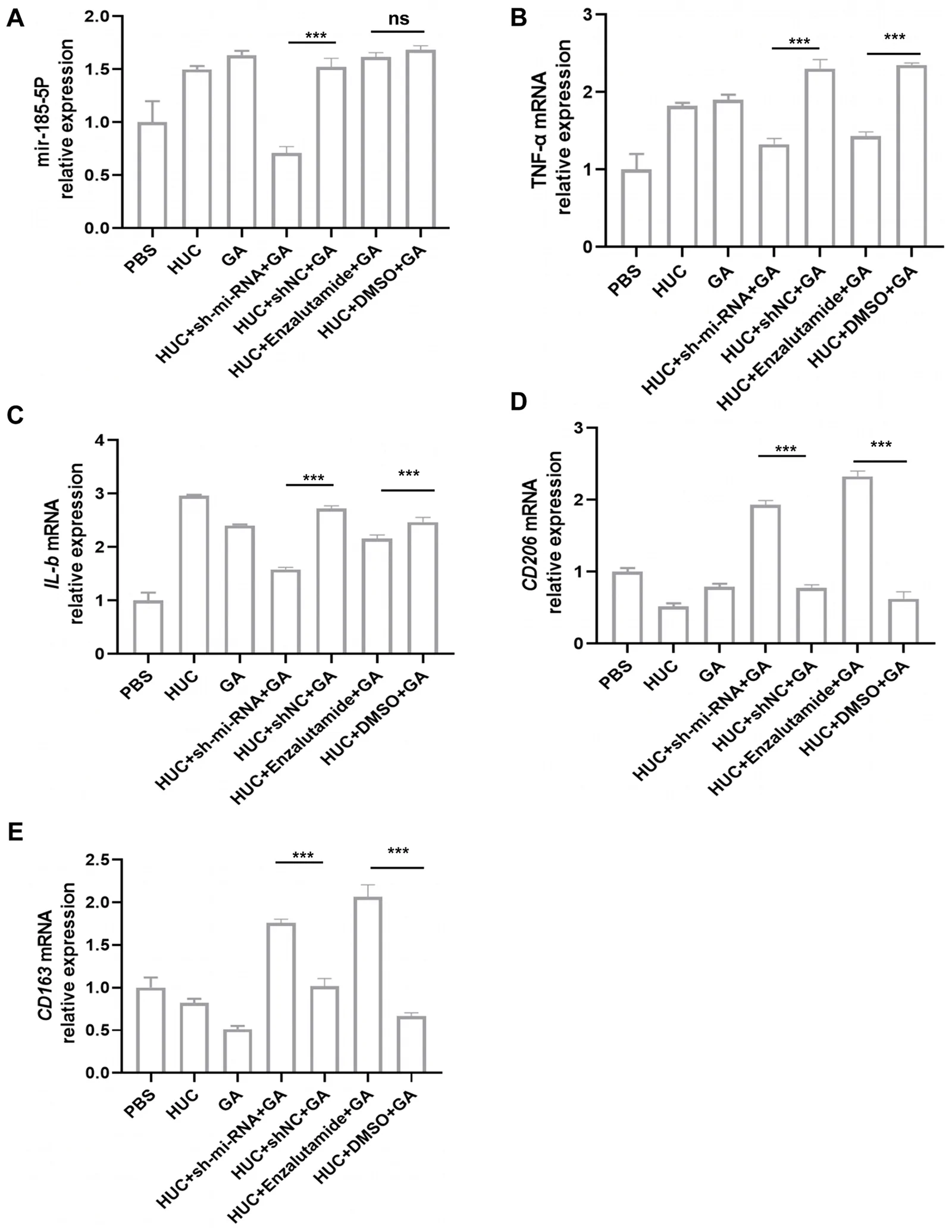
miR-185-5p is a key factor in AR-mediated regulation of TGF-β expression. (A) qPCR validation of miR-185-5p expression. (B) qPCR analysis of the effects of enzalutamide treatment or miR-185-5p expression on TNF-α expression. (C) qPCR analysis of the effects of enzalutamide treatment or miR-185-5p expression on IL-1β expression. (D) qPCR analysis of the effects of enzalutamide treatment or miR-185-5p expression on CD206 expression. (E) qPCR analysis of the effects of enzalutamide treatment or miR-185-5p expression on CD163 expression. ns, not significant; ***, *P* < 0.001.

## Discussion

Kidney stone disease (nephrolithiasis) is a prevalent and recurrent urological disorder associated with severe pain, renal injury, and a significant healthcare burden [1,22,23]. While hyperuricemia is a well-established metabolic risk factor for stone formation, the precise cellular and molecular mechanisms linking elevated uric acid to impaired crystal clearance within the kidney remain incompletely understood. This gap in knowledge hinders the development of targeted therapeutic strategies aimed at preventing stone recurrence.

This study systematically investigates a novel signaling axis by which uric acid stimulation of renal tubular epithelial cells dysregulates the local immune microenvironment to promote crystal deposition. We demonstrate that uric acid upregulates the AR, and this was accompanied by upregulation of miR-185-5p to suppress TGF-β secretion. This epithelial cell-derived signal impairs macrophage recruitment, M2 polarization, and phagocytic function, ultimately facilitating renal crystal deposition.

This AR/miR-185-5p/TGF-β axis represents a significant departure from the traditional view of hyperuricemia as a purely metabolic disorder, positioning it as a modulator of epithelial cell paracrine signaling. The identification of AR, a receptor more commonly associated with sex hormone signaling, as a key sensor for a metabolic stressor like uric acid expands our understanding of its pathophysiological roles in non-reproductive tissues [24–27]. This pathway provides a mechanistic explanation for how sustained high uric acid levels, a known risk factor for nephrolithiasis and cardiovascular complications [28–31], can transduce a signal that disrupts the local renal microenvironment, thereby creating a permissive niche for crystal retention and growth. The involvement of miR-185-5p aligns with emerging evidence highlighting the role of non-coding RNAs in the pathogenesis of kidney stone disease [32–35], and our data pinpoint a specific miRNA-effector relationship within the context of hyperuricemia.

At the cellular and functional level, the dysregulation of this epithelial signaling axis has profound consequences on macrophage behavior, a critical determinant of crystal fate [15,36–38]. The reduction in TGF-β secretion from stressed tubular cells impairs the recruitment and functional polarization of macrophages towards an anti-inflammatory, pro-repair M2 phenotype [39–42]. Our co-culture and in vivo data demonstrate that this shift away from M2 polarization, characterized by decreased expression of markers like ARG1 and CD206, is coupled with a significant deficit in the phagocytic clearance of crystals. This establishes a link between epithelial metabolic stress and innate immune dysfunction in the kidney. Macrophages are plastic cells whose polarization state (M1/M2) dictates their role in inflammation and tissue repair [43–46]. The failure to adequately polarize towards an M2 state in response to crystal deposition likely perpetuates a low-grade inflammatory milieu and compromises tissue repair mechanisms, facilitating the aggregation and anchoring of crystals to the renal papilla. This process mirrors findings in other models of crystal nephropathy, where macrophage polarization dynamics influence disease outcomes. Thus, our results reframe kidney stone pathogenesis as not merely a physicochemical process of supersaturation but as a failure of the epithelial-immune dialogue necessary for maintaining renal sterility against crystalline aggregates. The biological implications of changes in TGF-β require cautious interpretation because this cytokine participates in both immune regulation and renal fibrosis. An increase in TGF-β should therefore not be interpreted as uniformly beneficial. Further studies are needed to determine its net effects on inflammation, crystal deposition, and fibrosis in this setting.

The immunological implications of this disrupted crosstalk are central to the pathogenesis of hyperuricemia-associated nephrolithiasis. The impaired M2 macrophage response signifies a breakdown in the kidney’s intrinsic defense mechanism against particulate deposits. M2 macrophages are instrumental in resolving inflammation, promoting fibrosis regression, and engaging in efferocytosis; their functional deficiency creates an environment where initial crystal nuclei are not efficiently removed [47–50]. This allows micro-crystals to persist, grow, and potentially erode into the renal interstitium, triggering further injury and inflammatory cycles. This mechanism may explain the clinical observation that not all individuals with hyperuricemia develop gout or stones, as individual variations in this epithelial-immune regulatory pathway could confer differential susceptibility. Furthermore, the successful amelioration of crystal deposition and renal injury through pharmacological AR antagonism with Enzalutamide or genetic inhibition of miR-185-5p in vivo validates this pathway as a therapeutically targetable immune-metabolic node. Targeting this axis to restore protective macrophage function offers a novel strategy that moves beyond simply lowering serum uric acid levels, addressing instead the dysfunctional tissue response that enables stone formation, a concept gaining traction in understanding hyperuricemia’s systemic effects.

Several limitations warrant acknowledgment in interpreting our findings. First, while clinical correlations were established using urinary biomarkers, the core mechanistic insights rely on cell lines and rodent models; direct validation of the AR/miR-185-5p/TGF-β axis and associated macrophage phenotypes in human renal biopsy specimens from hyperuricemic stone formers remains absent. Second, the HK-2/THP-1 co-culture system, though informative, simplifies the complex renal microenvironment by excluding critical interactions with fibroblasts, endothelial cells, and other immune subsets that may modulate this pathway. Third, the chemically induced rat model may not fully recapitulate the chronic, multifactorial etiology of human nephrolithiasis, and the exclusive use of male subjects precludes an assessment of sex-specific differences mediated by androgen signaling. Finally, while we demonstrate an inverse correlation between miR-185-5p and TGF-β, definitive proof of direct targeting via luciferase reporter assays and exploration of potential off-target effects of miR-185-5p are needed to solidify the molecular hierarchy.

In conclusion, the present data suggest that an AR/miR-185-5p/TGF-β signalling pattern may contribute to altered epithelial–macrophage crosstalk and renal crystal deposition in hyperuricemic experimental models. Future research should prioritize validating this pathway in human tissues, developing renal-specific delivery systems for miR-185-5p antagonists, and exploring its broader relevance in other crystallopathies and chronic kidney diseases to translate these mechanistic insights into clinical practice.

## Supporting information

S1 File This PDF contains all the images of Western blotting. (PDF)

S2 File This zip file contains the original data and experimental reports for the WB experiment, PCR experiment and uric acid content detection. (ZIP)

S3 File This zip file contains the pictures and the experimental report of the migration assay. (ZIP)

S4 File This zip file contains the pictures and the experimental report of the migration assay. (ZIP)

S5 File This zip file contains the pictures and the experimental report of the migration assay. (ZIP)
