# Supplementary material for: The AR/miR-185-5p/TGF-β axis in renal tubular epithelial cells may regulate hyperuricemia-induced renal crystal deposition by impairing macrophage recruitment and M2 polarization
Source: PLoS One. 2026 Nov 11;21(11):e0360746. doi: 10.1371/journal.pone.0360746 (PMC13754159; doi:10.1371/journal.pone.0360746)
Supplement: Supplementary file 1 [file pone.0360746.s001.pdf]

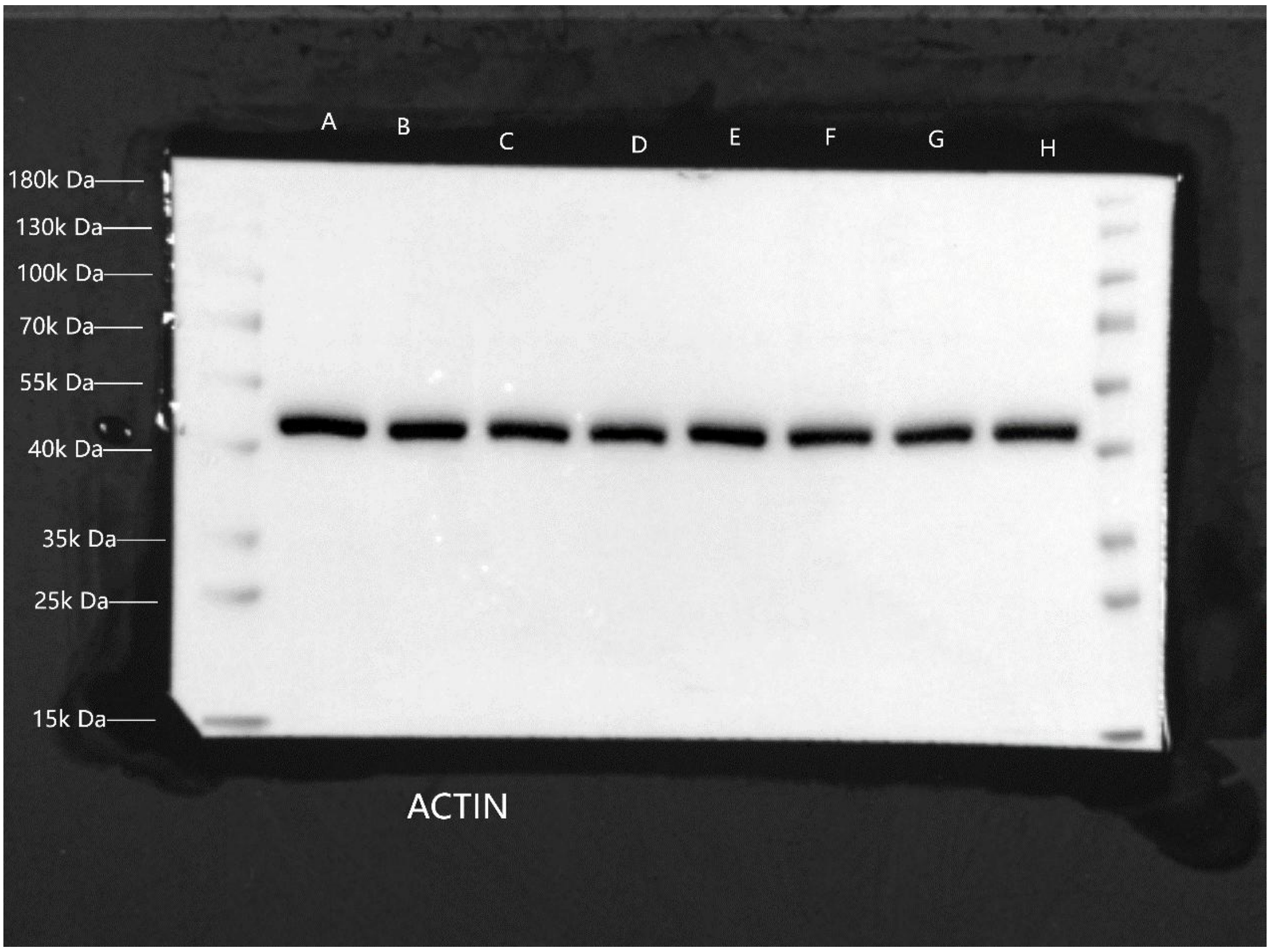
A
B
C
D
E
F
G
H
180k Da
130k Da
100k Da
70k Da
55k Da
40k Da
35k Da
25k Da
15k Da
ACTIN

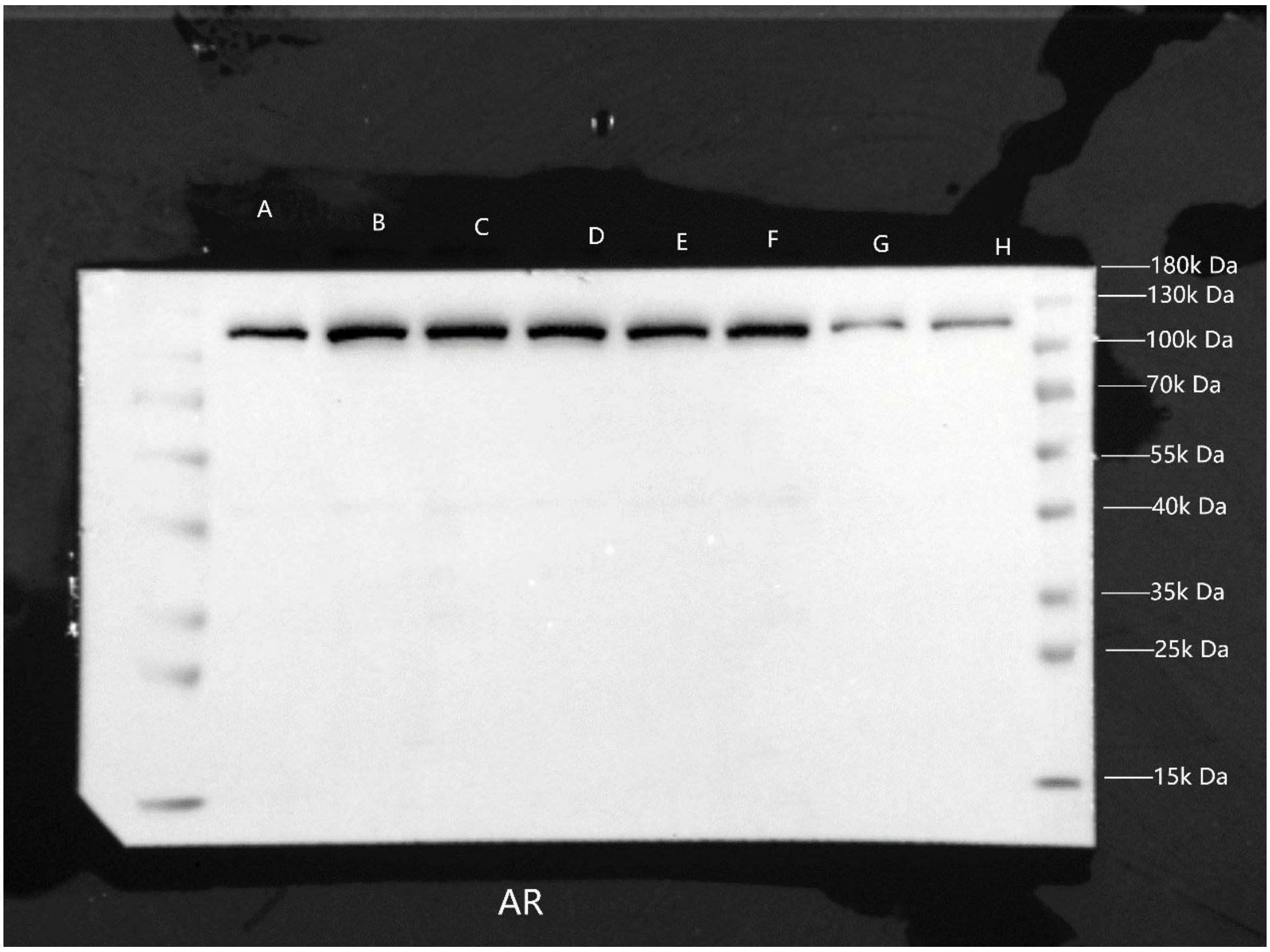
A
B
C
D
E
F
G
H
180k Da
130k Da
100k Da
70k Da
55k Da
40k Da
35k Da
25k Da
15k Da
AR

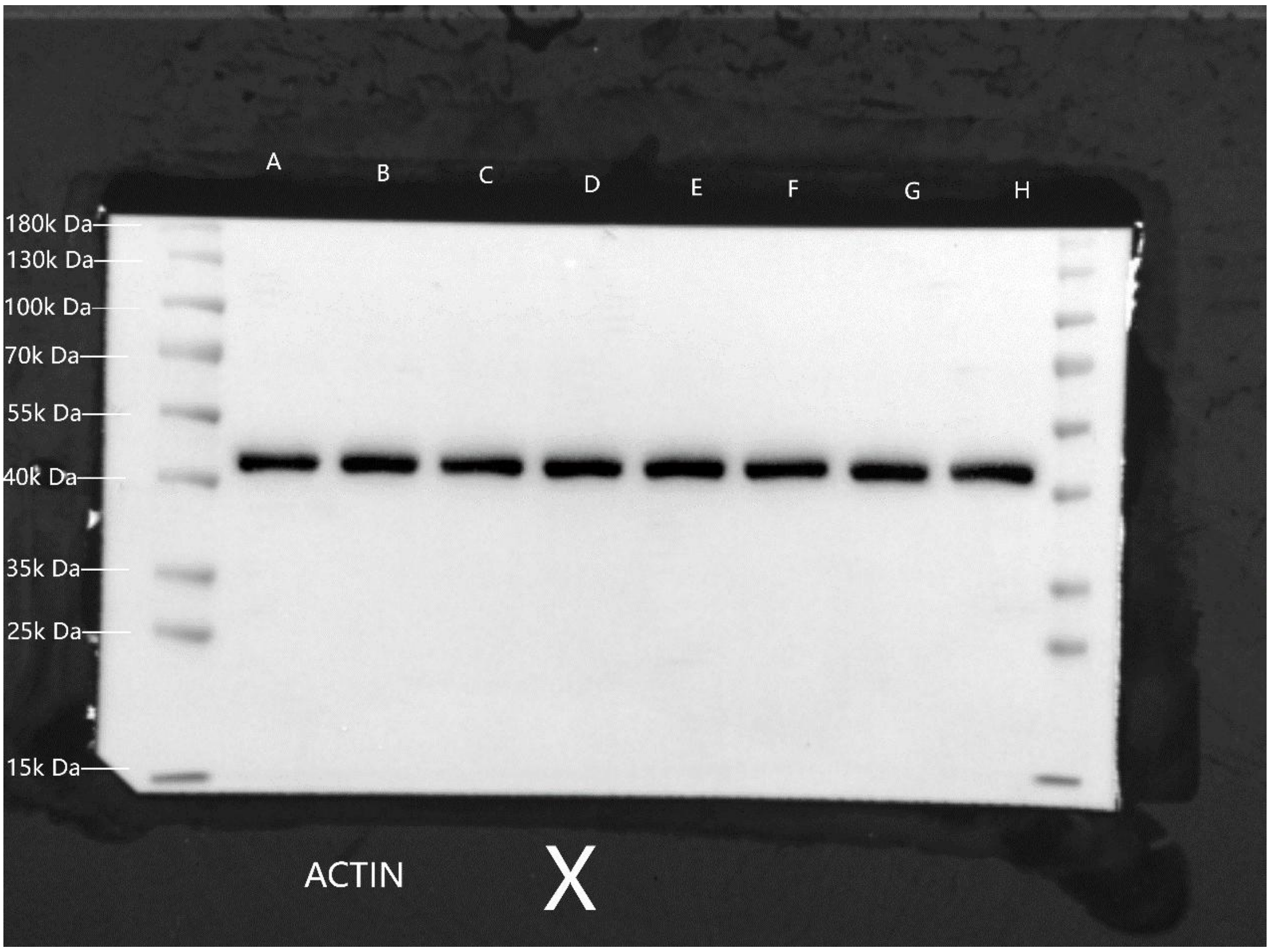
A
B
C
D
E
F
G
H
180k Da
130k Da
100k Da
70k Da
55k Da
40k Da
35k Da
25k Da
15k Da
ACTIN
X

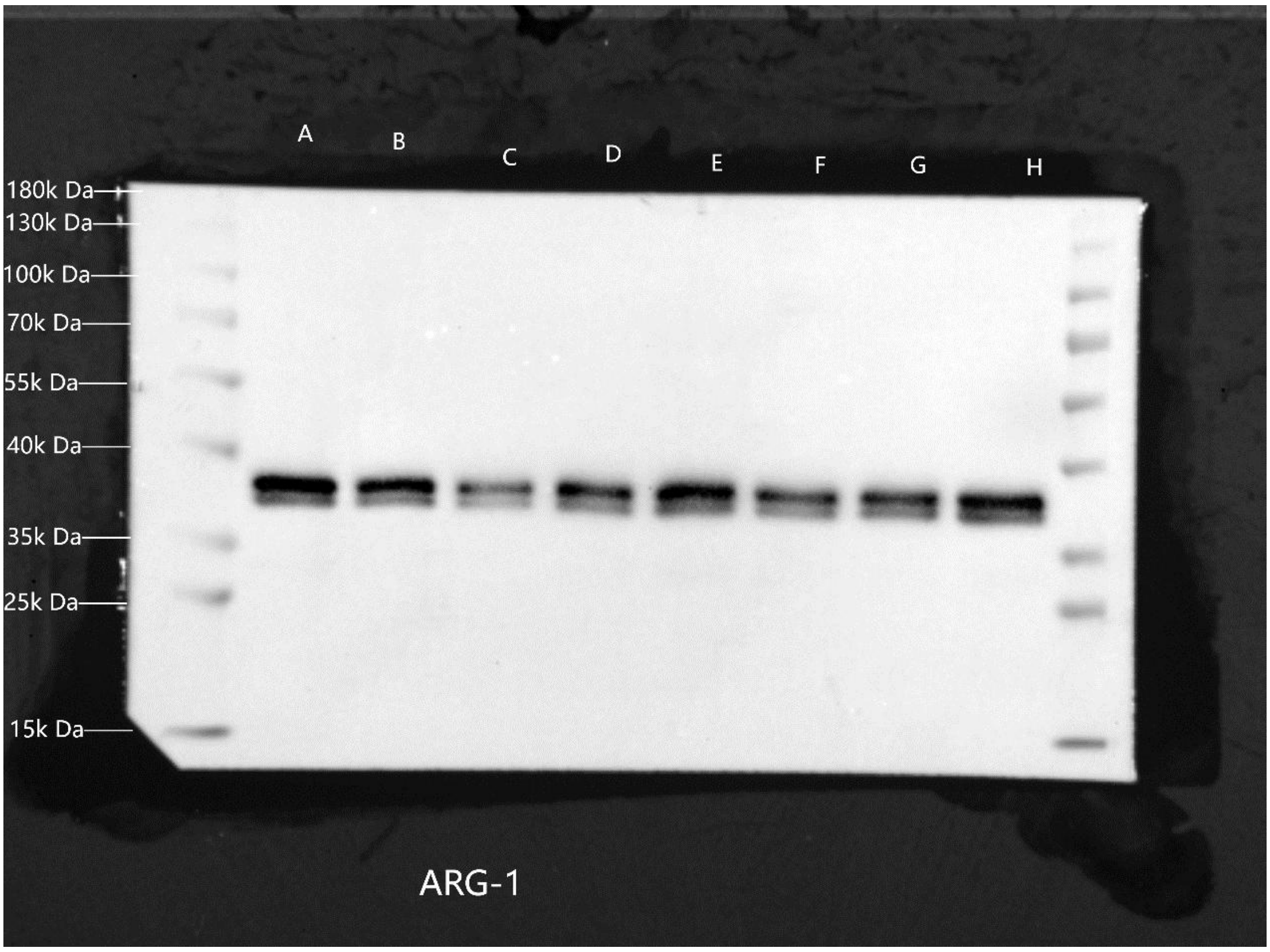
A
B
C
D
E
F
G
H
180k Da
130k Da
100k Da
70k Da
55k Da
40k Da
35k Da
25k Da
15k Da
ARG-1

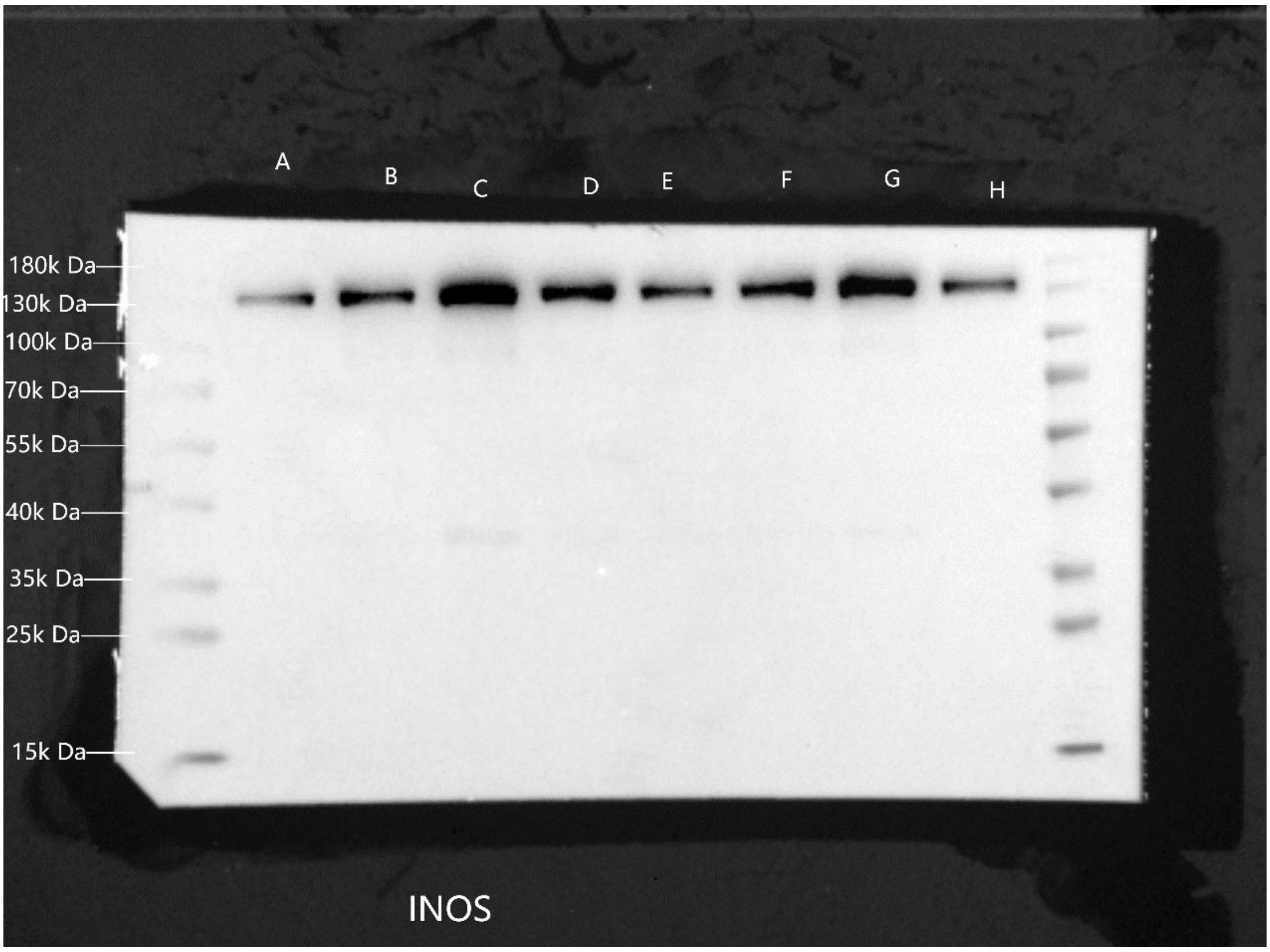

A: PBS; B: HUC; C: mir-185-5p mincs + HUC; D: mir-185-5p mincs NC + HUC; E: mir-185-5p inhibitor + HUC; F: mir-185-5p inhibitor NC + HUC; G: shAR+ mir-185-5p mincs + HUC; H: shAR+ mir-185-5p mincs NC + HUC

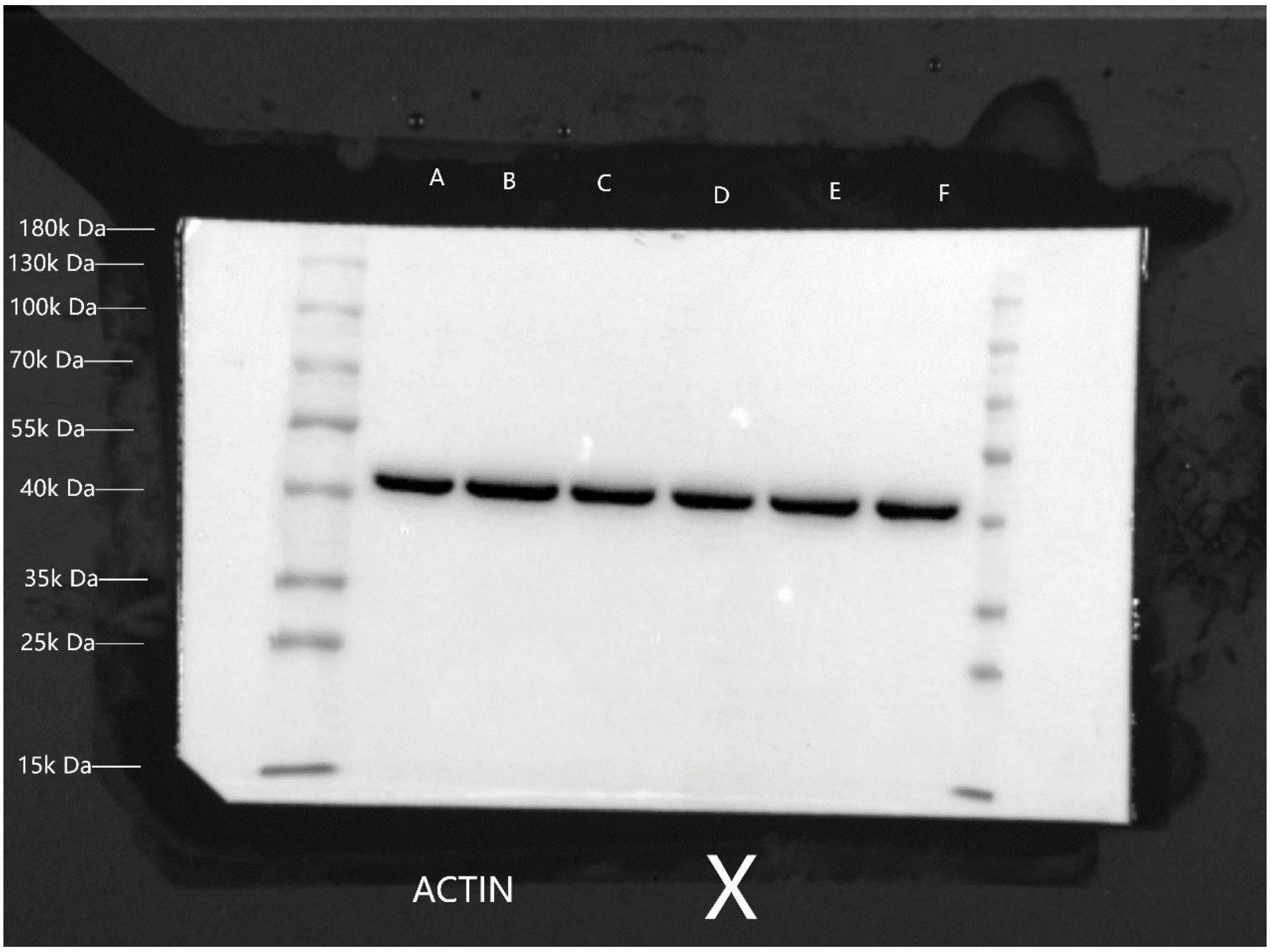
A
B
C
D
E
F
180k Da
130k Da
100k Da
70k Da
55k Da
40k Da
35k Da
25k Da
15k Da
ACTIN
X

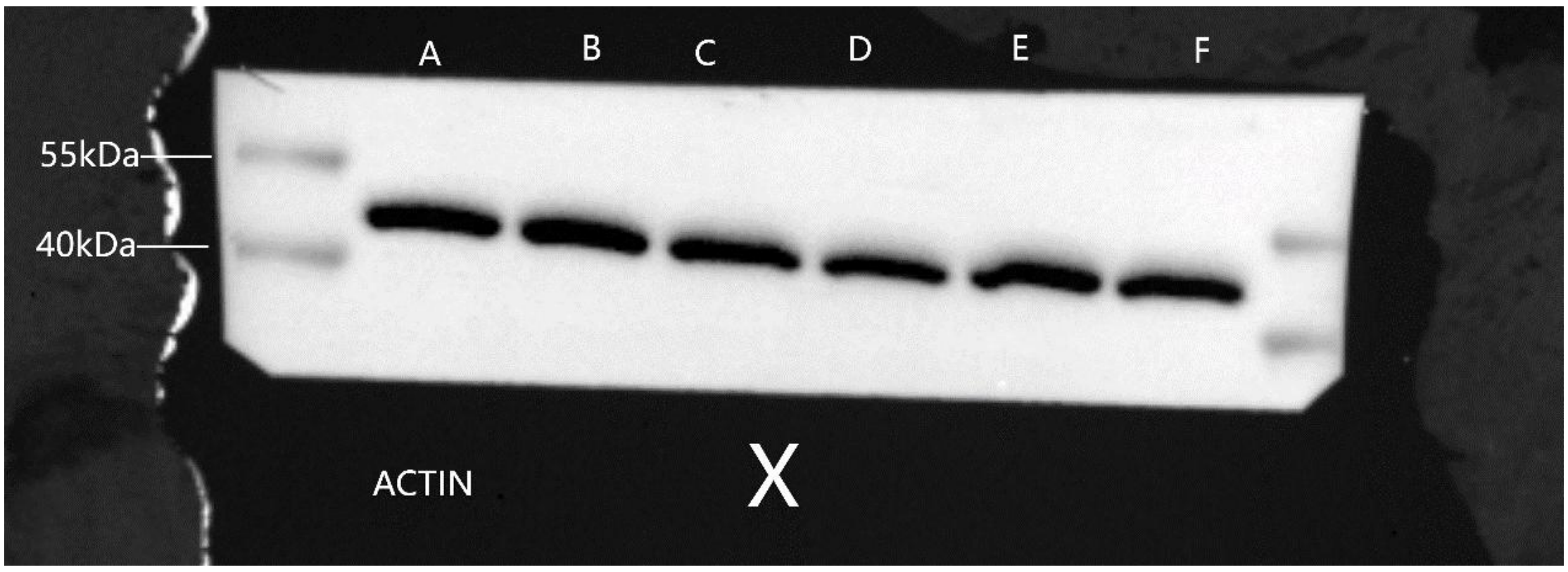
A
B
C
D
E
F
55kDa
40kDa
ACTIN
X

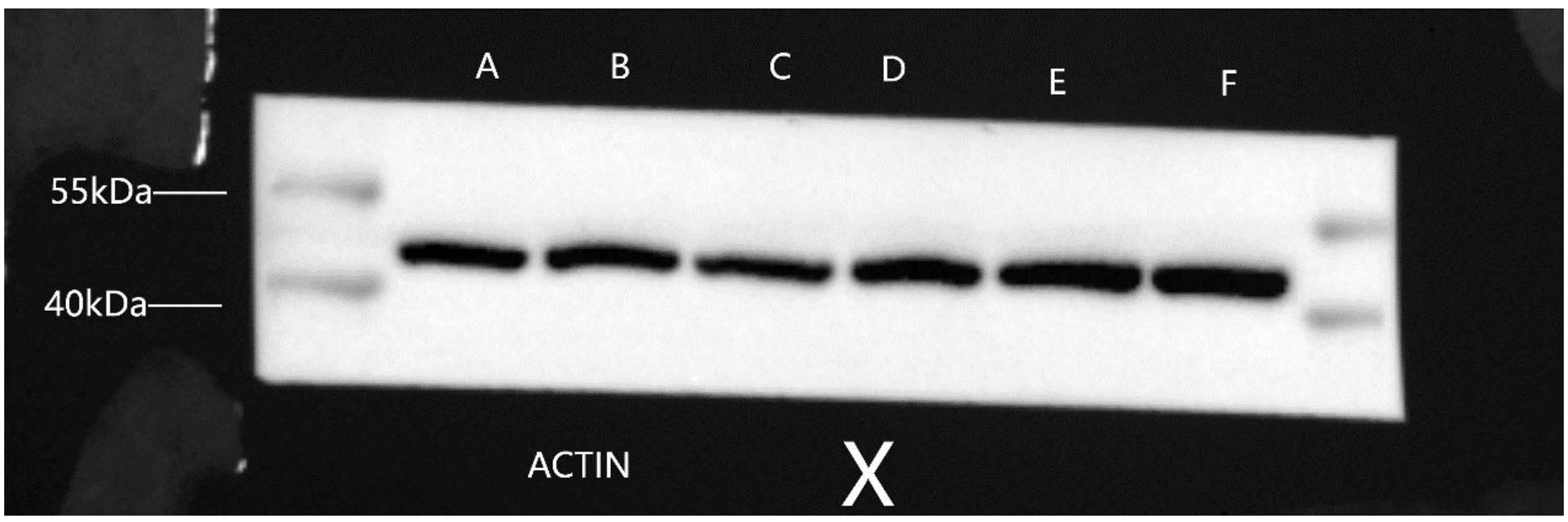
A
B
C
D
E
F
55kDa
40kDa
ACTIN
X

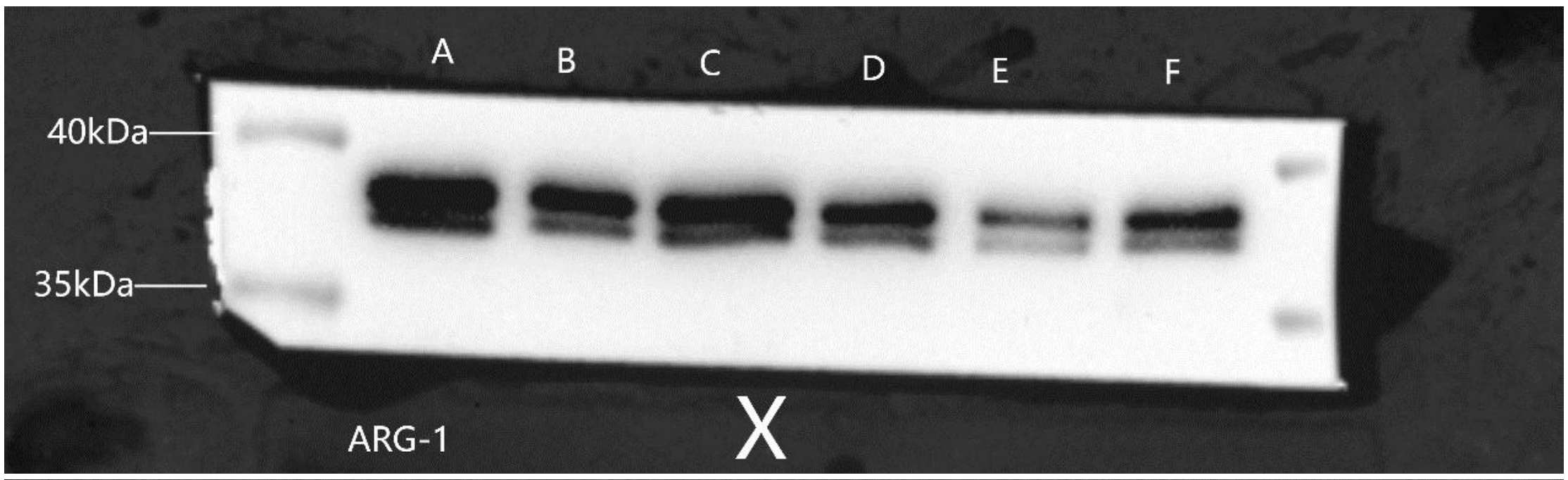
A
B
C
D
E
F
40kDa
35kDa
ARG-1
X

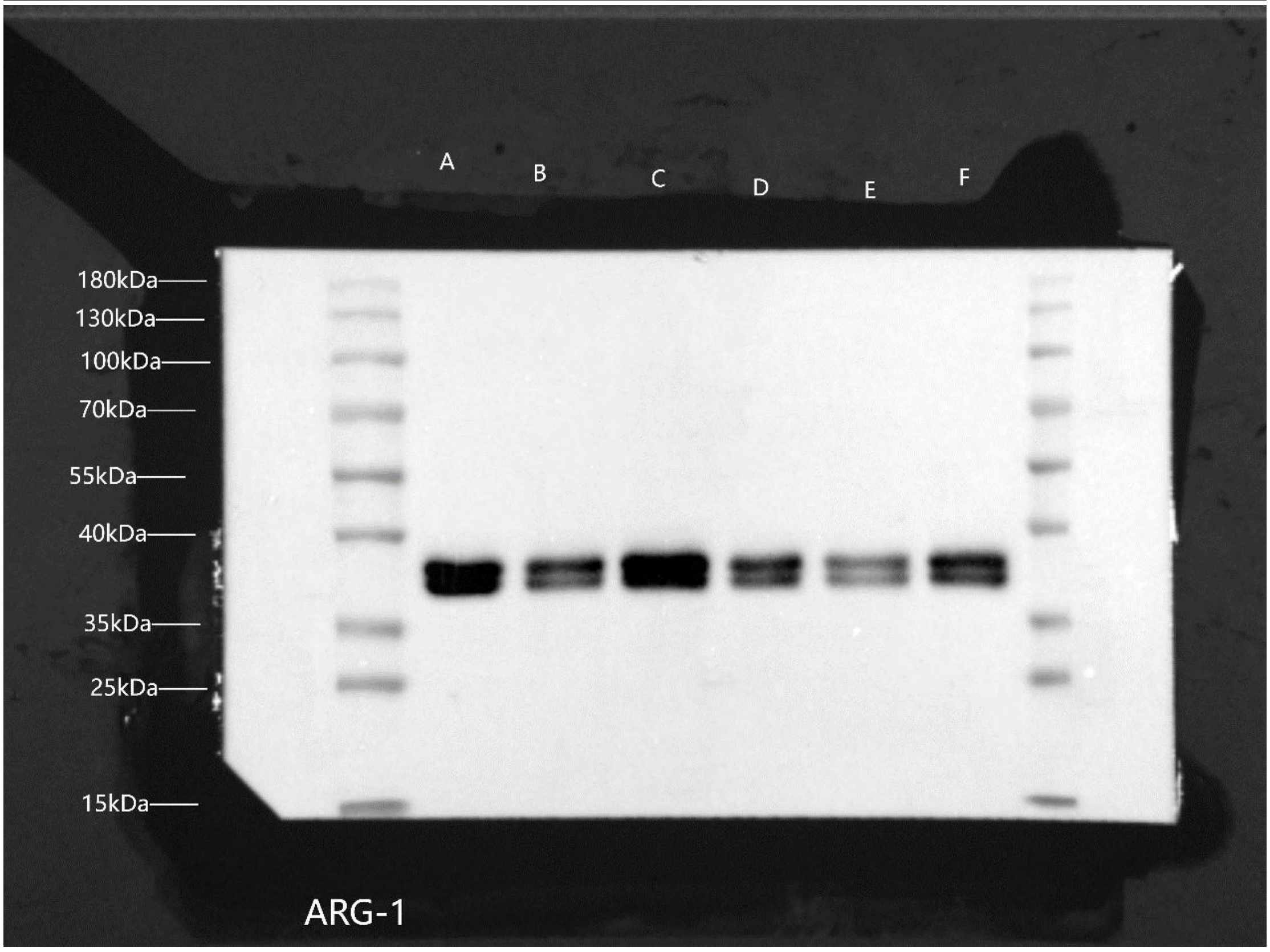
A
B
C
D
E
F
180kDa
130kDa
100kDa
70kDa
55kDa
40kDa
35kDa
25kDa
15kDa
ARG-1

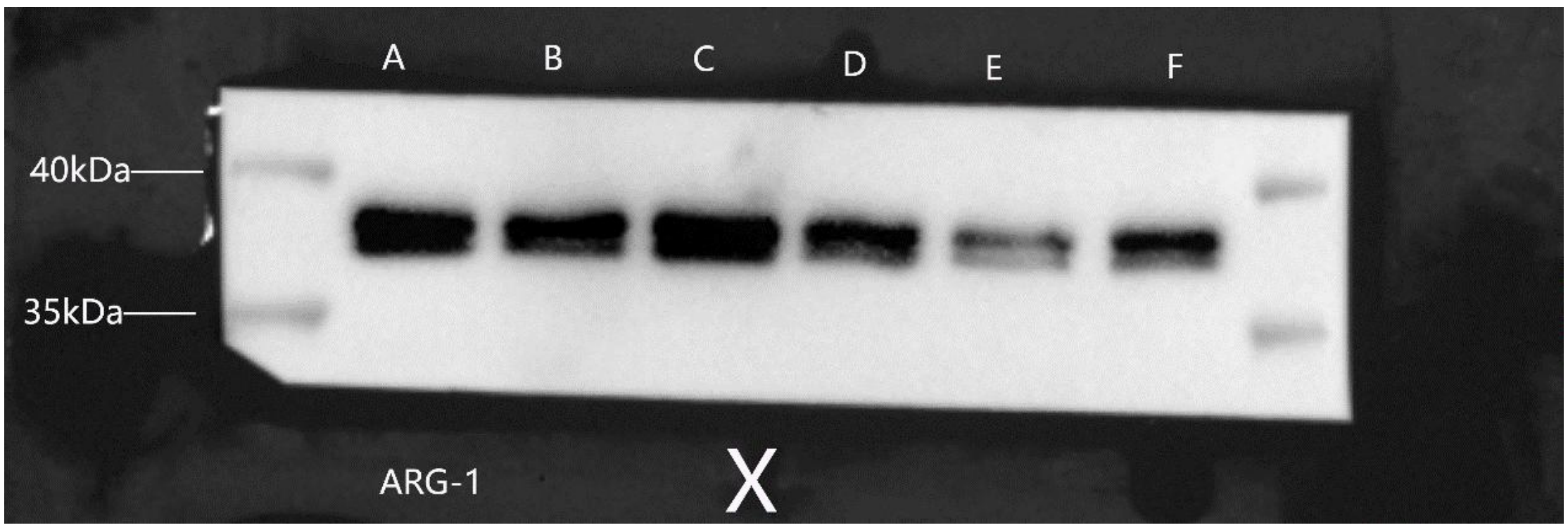
A
B
C
D
E
F
40kDa
35kDa
ARG-1
X

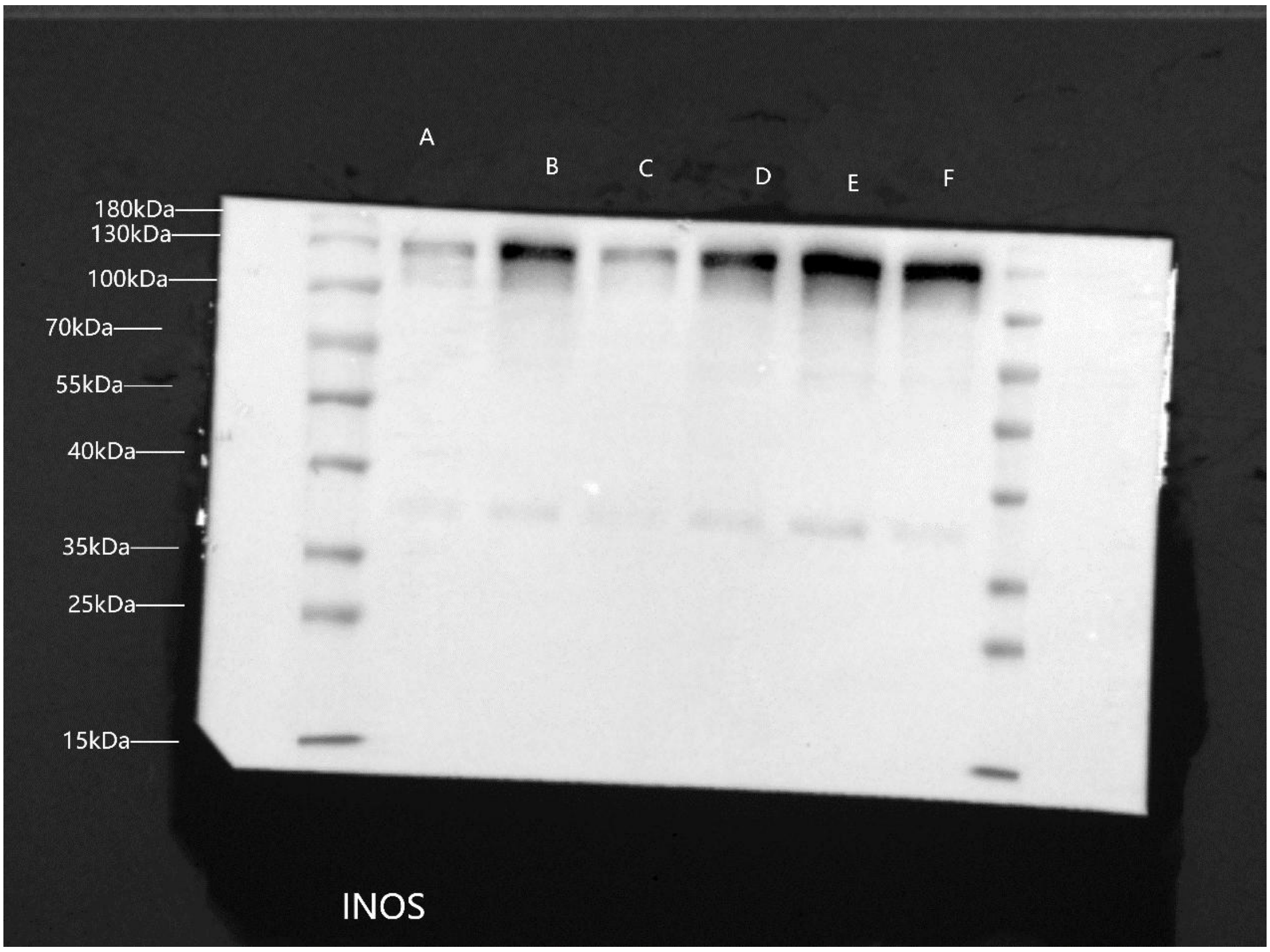
A
B
C
D
E
F
180kDa
130kDa
100kDa
70kDa
55kDa
40kDa
35kDa
25kDa
15kDa
INOS

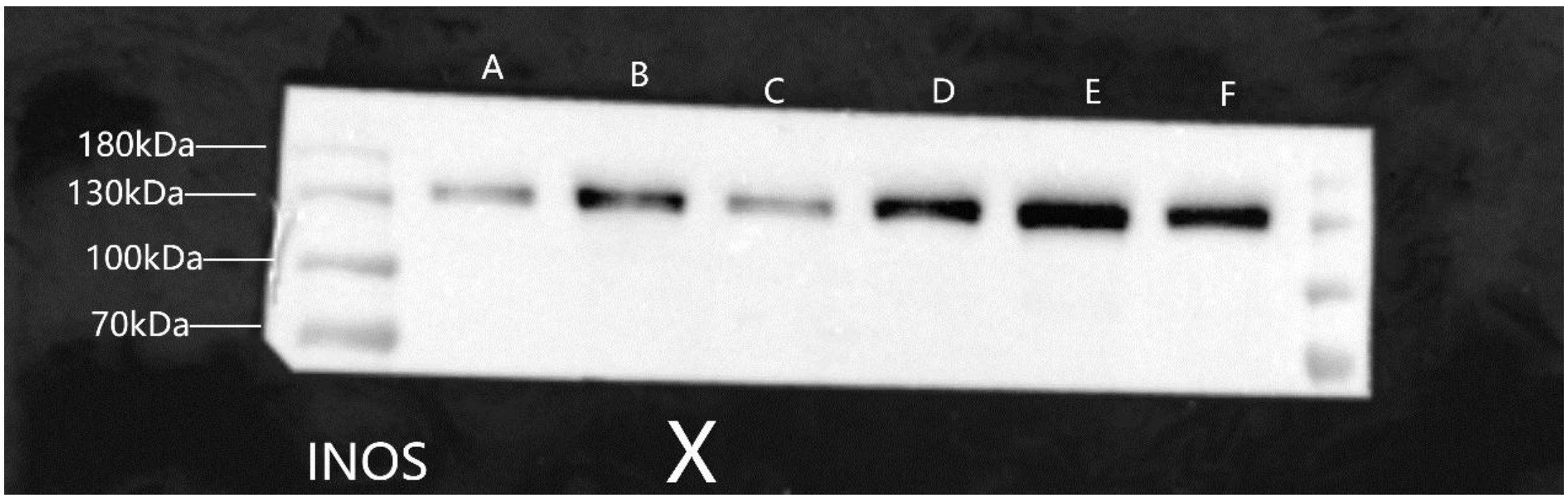
A
B
C
D
E
F
180kDa
130kDa
100kDa
70kDa
INOS
X

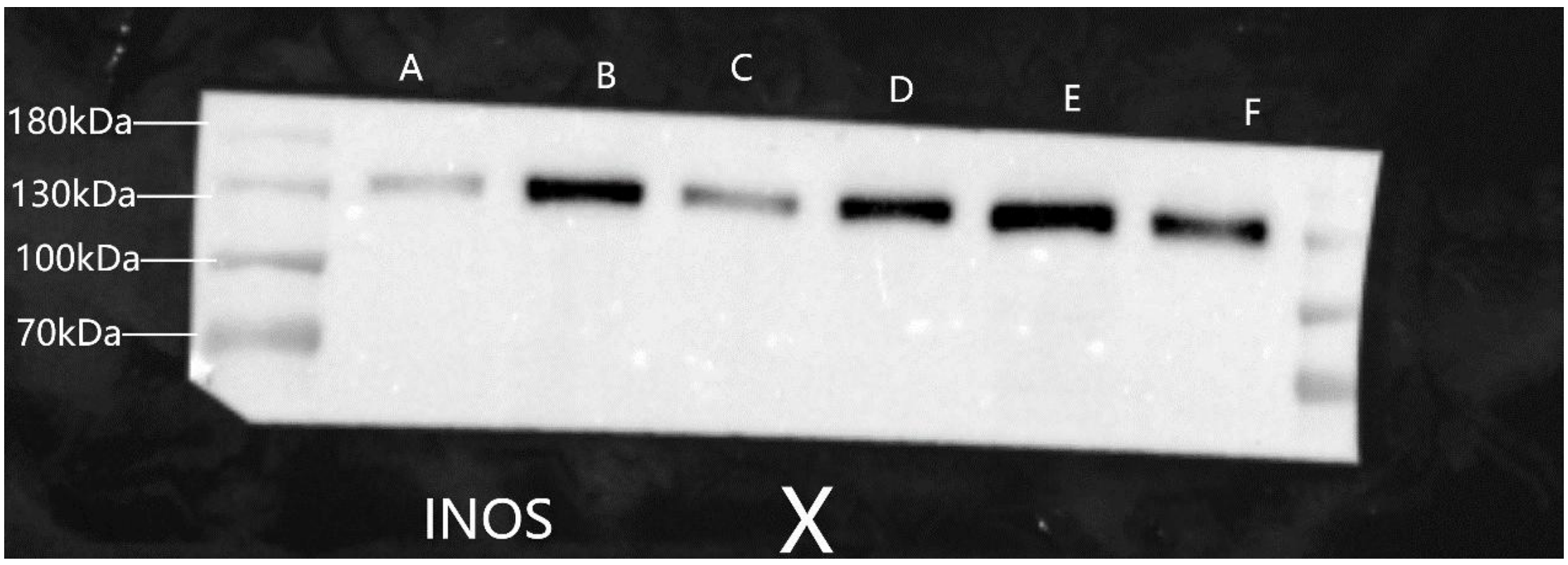
A
B
C
D
E
F
180kDa
130kDa
100kDa
70kDa
INOS
X

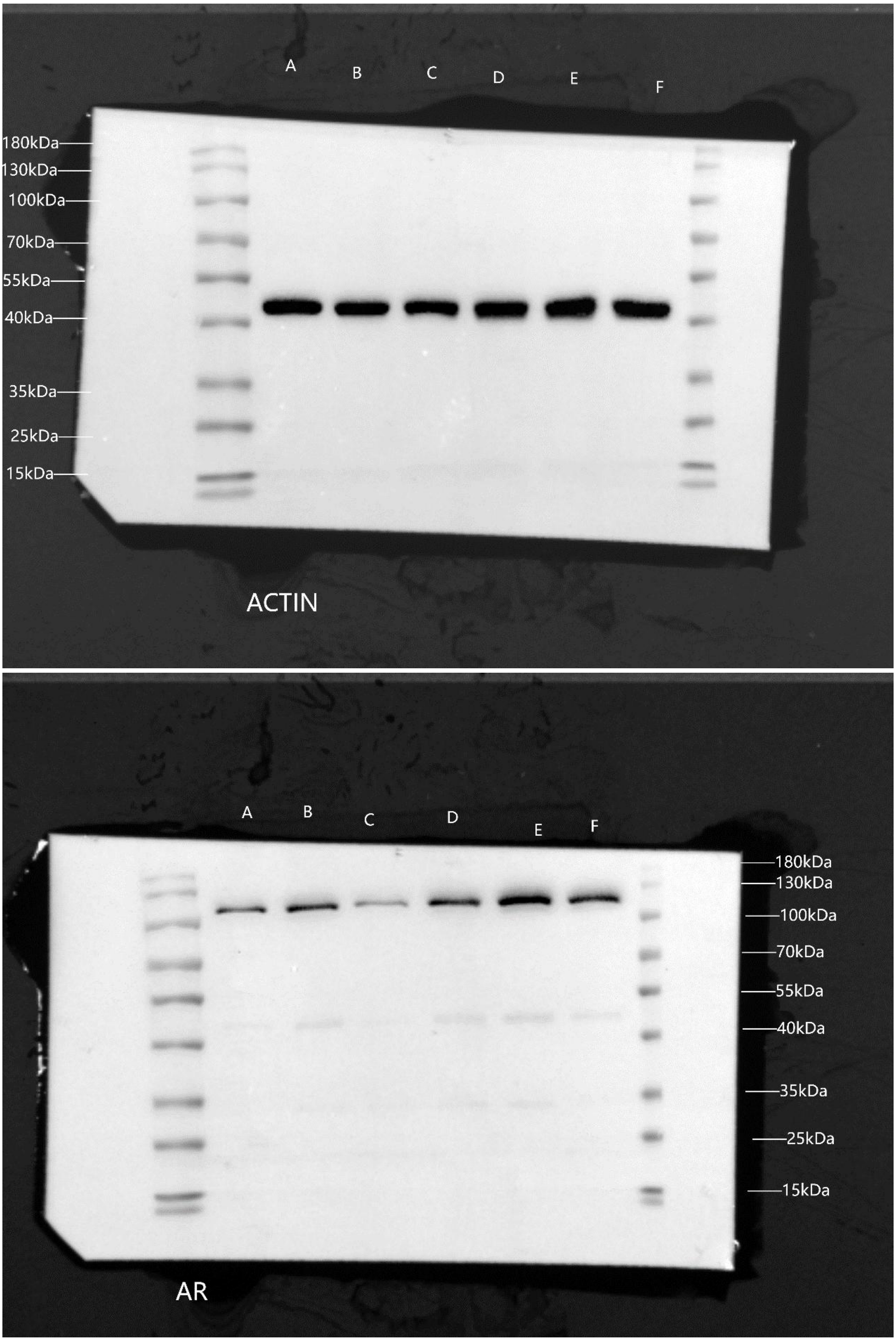

A: CON; B: HUC; C: shAR+HUC; D: shNC+HUC; E: OE-AR+HUC; F: OE-NC+HUC

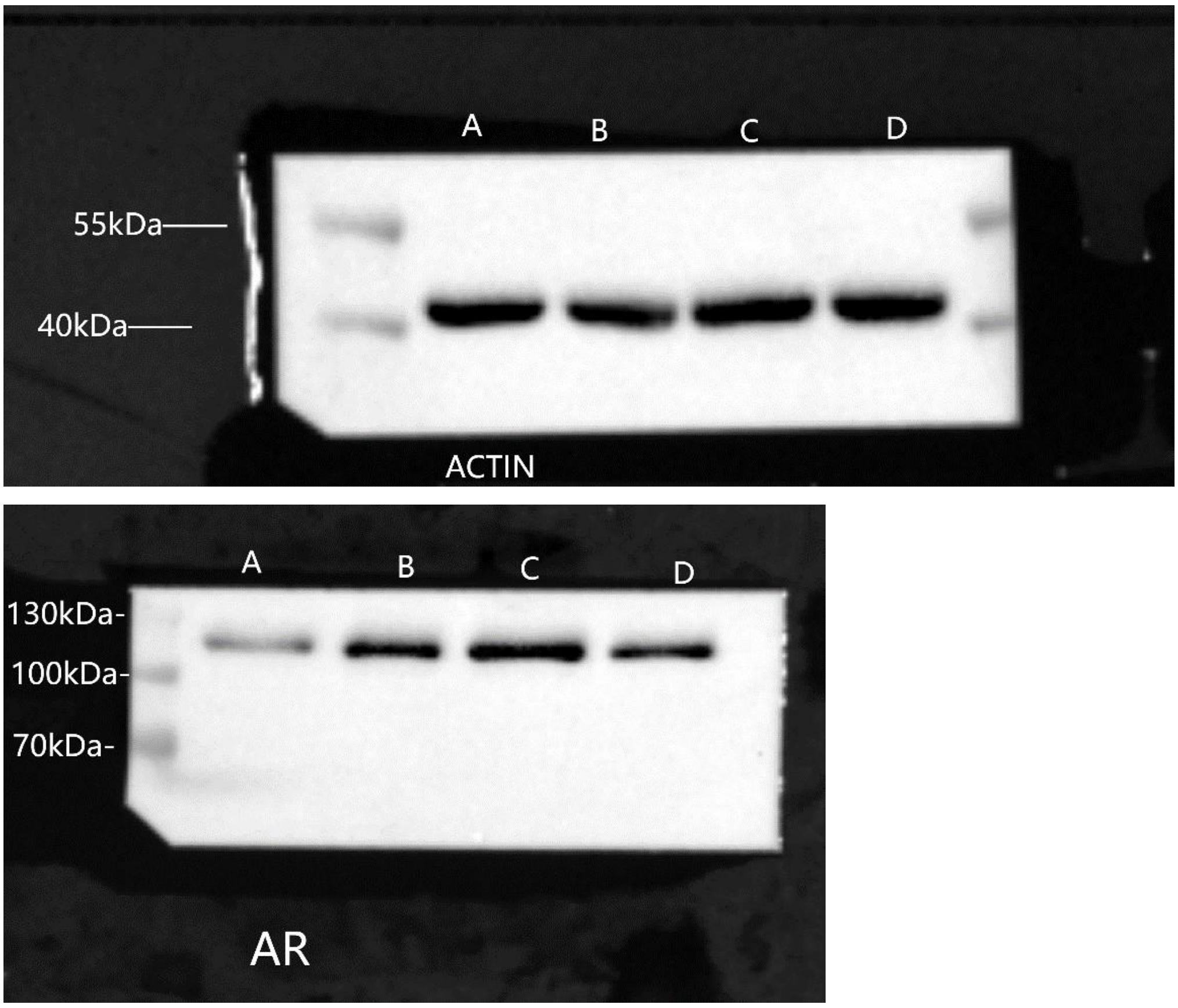

A:HK2-KD; B:HK2-NC; C:HK2-OE; D:HK2-vector;
