# Supplementary material for: The AR/miR-185-5p/TGF-β axis in renal tubular epithelial cells may regulate hyperuricemia-induced renal crystal deposition by impairing macrophage recruitment and M2 polarization
Source: PLoS One. 2026 Nov 11;21(11):e0360746. doi: 10.1371/journal.pone.0360746 (PMC13754159; doi:10.1371/journal.pone.0360746)
Supplement: Supplementary file 2 [file pone.0360746.s002.zip › Supporting Information 1/250829 3.5Western blotting/lab report.docx]

**Western Blot Laboratory Report**

# **1. Experimental Materials**

### **1.1 Equipment**

**Table 1. Main equipment**

| **Item** | **Manufacturer** | **Model** |
| --- | --- | --- |
| Electrophoresis power supply | Beijing Liuyi Instrument Factory | DYY-7C |
| Vertical electrophoresis tank | Beijing Liuyi Instrument Factory | DYCZ-24DN |
| Electrotransfer apparatus | Beijing Liuyi Instrument Factory | DYCZ-40 |
| Microplate reader | BioTek | EPOCH2 |

### **1.2 Main Reagents**

**Table 2. Main reagents**

| **Reagent** | | **Manufacturer** | **Catalog No.** |
| --- | --- | --- | --- |
| Phosphatase inhibitor | | Beyotime | S1873 |
| PMSF | | Beyotime | ST506 |
| RIPA lysis buffer | | Beyotime | P0013B |
| BCA protein assay kit | | Beyotime | P0010 |
| TEMED | | Amresco | Amresc00761 |
| SDS loading buffer | | Beyotime | P0015L |
| 30% acrylamide | | Solarbio | A1010 |
| TG | Tris-base | BioFROXX | 1115 |
|  | Glycine | BioFROXX | 12751 |
|  | SDS | BioFROXX | 3250 |
| Transfer buffer | Tris-base | BioFROXX | 1115 |
|  | Glycine | BioFROXX | 12751 |
|  | Methanol | Sinopharm Chemical Reagent Co., Ltd. | 10014118 |
| Protein marker (10–180 kDa) | | Thermo Fisher Scientific | 26616 |
| Nitrocellulose (NC) membrane | | Pall | 66485 |
| ECL substrate solution | | Thermo | NCI5079 |

**2. Experimental Procedures**

**2.1 Sample Preparation**

Wash the cells twice with prechilled PBS and discard the wash solution. Place the cells on ice and add 150 μL of lysis buffer. Scrape the cells with a clean cell scraper and transfer them to a 1.5 mL centrifuge tube. Disrupt the cells by sonication and incubate on ice for 30 min to complete lysis. Centrifuge at 12,000 rpm for 15 min at 4 °C and collect the supernatant. Prepare the BCA working solution and measure the protein concentration of each sample. Add loading buffer at a sample-to-loading-buffer ratio of 4:1. Heat at 95 °C for 8 min, allow to cool, and store at 4 °C.

**2.2 SDS–PAGE**

Prepare a 10% resolving gel and a 5% stacking gel. Load 20 μL per well. Run electrophoresis at a constant voltage of 80 V until the bromophenol blue dye enters the resolving gel from the stacking gel. Increase the voltage to 100 V and continue until the dye reaches 1.5 cm from the bottom of the gel.

**2.3 Membrane Transfer**

Prepare the transfer buffer. Place the NC membrane, filter paper, and gel removed after SDS–PAGE in a tray containing transfer buffer. Assemble the transfer sandwich in the following order: sponge, three layers of filter paper, resolving gel, NC membrane, three layers of filter paper, and sponge. Transfer in the transfer tank at a constant current of 300 mA.

**2.4 Blocking**

Remove the NC membrane and block with 5% skim milk for 1 h at room temperature on a shaker.

**2.5 Primary Antibody Incubation**

Prepare the primary antibody solutions at the appropriate dilutions. Incubate the membrane in the primary antibody solution overnight at 4 °C.

| **Target** | **Manufacturer** | **Catalog No.** | **Dilution** | **Host** | **Molecular weight** |
| --- | --- | --- | --- | --- | --- |
| AR | Affinity | DF6783 | 1:1000 | Rabbit | 110kDa |
| β-actin | Proteintech | 66009-1-Ig | 1:50000 | Mouse | 42kDa |

**2.6 Secondary Antibody Incubation**

Wash the membrane four times with TBST for 5 min each. Prepare the secondary antibody solutions and incubate the membrane in the secondary antibody solution for 1 h at room temperature.

| **Secondary antibody** | **Catalog No.** | **Dilution** |
| --- | --- | --- |
| HRP-conjugated Affinipure Goat Anti-Rabbit IgG(H+L) | SA00001-2 | 1:5000 |
| HRP-conjugated Affinipure Goat Anti-Mouse IgG(H+L) | SA00001-1 | 1:5000 |

**2.7 Chemiluminescent Detection**

Wash the membrane four times with TBST for 5 min each. Prepare the chemiluminescent substrate by mixing equal volumes of solutions A and B from the ECL kit. Apply the ECL substrate to the membrane, place the membrane in the imaging system, and acquire chemiluminescence images.
