# Supplementary material for: The AR/miR-185-5p/TGF-β axis in renal tubular epithelial cells may regulate hyperuricemia-induced renal crystal deposition by impairing macrophage recruitment and M2 polarization
Source: PLoS One. 2026 Nov 11;21(11):e0360746. doi: 10.1371/journal.pone.0360746 (PMC13754159; doi:10.1371/journal.pone.0360746)
Supplement: Supplementary file 2 [file pone.0360746.s002.zip › Supporting Information 1/Experiment 2/250811 2.3PCR lab report/lab report.docx]

Real-Time Quantitative PCR Laboratory Report

**1. Experimental Materials**

**1.1 Equipment and Consumables**

**Table 1. Main equipment and consumables**

| **Item** | **Manufacturer** | **Model** |
| --- | --- | --- |
| Micropipette | Eppendorf |  |
| Forced-air drying oven | Shanghai Jinghong Laboratory Equipment Co., Ltd. | DHG 9203A |
| Real-time quantitative PCR instrument | ABI | StepOnePlus |
| qPCR tubes | Nest |  |
| Microvolume spectrophotometer | Hangzhou Allsheng Instruments Co., Ltd. | Nano-100 |
| Constant-temperature water bath | Beijing Changfeng Instrument Co., Ltd. | HW-SY11-K P2 |
| Thermal cycler | Dongsheng Innovation Biotechnology Co., Ltd. | EDC-810 |
| 1 mL RNase-free pipette tips (bagged) | EXTRAGENE |  |
| 200 μL RNase-free pipette tips (bagged) | EXTRAGENE |  |
| 10 μL RNase-free pipette tips (bagged) | EXTRAGENE |  |

**1.2 Main Reagents**

**Table 2. Main reagents**

| **Reagent or service** | **Manufacturer** | **Catalog No.** |
| --- | --- | --- |
| Trizol | Servicebio | G3013 |
| Chloroform substitute | Servicebio | G3014 |
| Reverse transcription kit | Novoprotein | E047 |
| 2×Universal Blue SYBR Green qPCR Master Mix | Servicebio | G3326 |
| DEPC-treated water | Servicebio | G3004 |
| Primer synthesis | Tsingke |  |

**2. Experimental Procedures**

**2.1 Primer Design and Synthesis**

**Table 3. Primer sequences**

| **Name** | **Primer** | **Sequence (5′–3′)** |
| --- | --- | --- |
| miR-185-5P | Forward | GCGCGGGAGAGAAAGGC |
|  | Reverse | AGTGCAGGGTCCGAGGTATT |
| miR-185-5P | Specific reverse transcription primer | GTCGTATCCAGTGCAGGGTCCGAGGTATTCGCACTGGATACGACTCGGCT |
| U6 | Forward | GCTTCGGCAGCACATATACTAAAAT |
|  | Reverse | CGCTTCACGAATTTGCGTGTCAT |
| ACTB | Forward | CATGTACGTTGCTATCCAGGC |
|  | Reverse | CTCCTTAATGTCACGCACGAT |

**2.2 RNA Extraction**

Adherent cells: Remove the culture medium and wash the cells with an appropriate volume of prechilled PBS. Aspirate the PBS as completely as possible. Add 1 mL of RNA extraction reagent per well of a 6-well plate or 0.5 mL per well of a 12-well plate. Gently rock the plate to ensure that the cells are fully exposed to the reagent. Pipette up and down to lyse the cells, transfer the lysate to a centrifuge tube, and incubate at room temperature for 5 min.

Suspension cells: Collect the cells by centrifugation and aspirate the supernatant as completely as possible. Add 1 mL of RNA extraction reagent per 1–10 × 10⁶ cells. Pipette up and down several times to lyse the cells, then incubate at room temperature for 5 min.

Tissue samples: Cut the tissue into small pieces (approximately soybean-sized) and place them in a prechilled homogenizer. Add 1 mL of RNA extraction reagent per 100 mg of tissue and homogenize until no visible tissue fragments remain. Optional: Centrifuge at 12,000 × g for 10 min at 4 °C to separate tissue debris, then transfer the supernatant to a fresh centrifuge tube.

Blood samples: Add 800 μL of RNA extraction reagent to each 200 μL of fresh blood. Mix thoroughly by repeated pipetting and incubate at room temperature for 5–10 min. For blood stored in tissue RNA stabilization solution (G3019), first centrifuge at 4,000–5,000 rpm for 5 min and discard the upper stabilization solution. Add 800 μL of RNA extraction reagent, mix thoroughly by repeated pipetting, and incubate at room temperature for 5–10 min.

① Add 100 μL of chloroform substitute (G3014) per 1 mL of RNA extraction reagent. Vortex (the SMV-4500B vortex mixer is recommended) or vigorously invert the tube for 15 s. Incubate at room temperature for 3–5 min.

② Centrifuge at 12,000 × g for 15 min at 4 °C. Carefully transfer the upper, colorless aqueous phase to a fresh centrifuge tube. Approximately 500–550 μL can be recovered per 1 mL of extraction reagent.

③ Precipitate the RNA: Add 500 μL of isopropanol to the collected aqueous phase and gently invert several times to mix. Incubate for 10 min at room temperature or 4 °C, or for 15 min at −20 °C. Centrifuge at 12,000 × g for 10 min at 4 °C. The white pellet at the bottom of the tube is total RNA. Discard the supernatant.

④ Add 1 mL of 75% ethanol and mix by inversion, preferably until the white pellet is suspended. Centrifuge at 12,000 × g for 5 min at 4 °C and discard the supernatant. Briefly centrifuge again at 5,000 × g for 3–5 s, then carefully remove all residual liquid with a fine pipette tip.

⑤ Leave the tube containing the RNA pellet open for 3–5 min to allow the pellet to air-dry briefly. Add 20–50 μL of DEPC-treated water or RNase-free water to dissolve the RNA completely. The RNA is then ready for concentration measurement and subsequent experiments. If subsequent experiments cannot be performed promptly, store the RNA at −70 °C.

**2.3 Reverse Transcription of RNA**

① Measure the concentration and purity of the extracted RNA using the Nano Bio 200. RNA with an A260/A280 ratio of 1.8–2.1 is considered sufficiently pure for reverse transcription.

**Table 4. RNA concentrations of the samples**

| **Sample** | **RNA concentration (ng/μL)** | **A260/A280** |
| --- | --- | --- |
| A: CON | 464.23 | 1.936 |
| B: Hyperuricemia | 538.90 | 1.967 |
| C: sh-AR + hyperuricemia | 520.67 | 1.955 |
| D: sh-NC + hyperuricemia | 678.46 | 1.978 |
| E: OE-AR + hyperuricemia | 631.04 | 1.983 |
| F: vector + hyperuricemia | 687.49 | 1.961 |

② Prepare the reverse transcription reaction mixture (20 μL).

**Table 5. Reverse transcription reaction mixture**

| Component | Volume |
| --- | --- |
| 2×cDNA Synthesis SuperMix | 10μL |
| gDNA Purge | 1μL |
| Template RNA | 1μg |
| RNase-free water | To 20 μL |

③ Set the thermal cycler program for reverse transcription to 50 °C for 15 min, followed by 85 °C for 5 s, then hold at 4 °C.

④ After reverse transcription, store the cDNA at −20 °C.

**2.4 Real-Time Quantitative PCR (ABI StepOnePlus)**

① Set up the real-time quantitative PCR instrument.

Preheat the ABI StepOnePlus real-time quantitative PCR instrument and launch the control software.

1. Enter the experiment name.

2. Specify the sample names and controls.

3. Assign the reaction wells.

4. Set the cycling program.

② Prepare the SYBR dye-based qPCR reaction mixture (20 μL).

**Table 6. qPCR reaction mixture**

| Component | Volume |
| --- | --- |
| 2×SYBR Green qPCR Master Mix | 10μL |
| Forward primer (10 μM) | 0.4μL |
| Reverse primer (10 μM) | 0.4μL |
| cDNA | 2μL |
| Nuclease-free water | 7.2μL |

③ Place the prepared reaction mixtures in the assigned wells of the ABI StepOnePlus real-time quantitative PCR instrument. Set the program to initial denaturation at 95 °C for 1 min, denaturation at 95 °C for 15 s, and annealing/extension at 60 °C for 1 min. Use the instrument’s default settings for the melting curve.

④ Run the reaction program.
