# Supplementary material for: The AR/miR-185-5p/TGF-β axis in renal tubular epithelial cells may regulate hyperuricemia-induced renal crystal deposition by impairing macrophage recruitment and M2 polarization
Source: PLoS One. 2026 Nov 11;21(11):e0360746. doi: 10.1371/journal.pone.0360746 (PMC13754159; doi:10.1371/journal.pone.0360746)
Supplement: Supplementary file 3 [file pone.0360746.s003.zip › Supporting Information 2/Migration Assay/Migration Assay Report.docx]

Transwell Migration Assay Report

## 1. Experimental Materials

## 1.1 Equipment

**Table 1. Main equipment**

| **Item** | **Manufacturer** | **Model** |
| --- | --- | --- |
| Micropipette | ThermoFisher |  |
| Clean bench | Shinva Medical | LPC-V-15B |
| CO₂ incubator | Thermo | 3111 |
| Inverted microscope | OLYMPUS | IX51 |
| Upright microscope | OLYMPUS | BX51 |
| Low-speed centrifuge | Eppendorf | 5702R |
| Microplate reader | Biotek | Epoch |

## 1.2 Main Reagents and Consumables

**Table 2. Main reagents**

| **Reagent** | **Manufacturer** | **Catalog No.** |
| --- | --- | --- |
| MEM | procell | PM150410 |
| 1640 | procell | PM150110 |
| Serum | GIBCO | 10099-141 |
| 0.25% Trypsin | Gibco | 15050065 |
| Lipo3000 | Thermos | L3000015 |
| Opti-MEN | Gibco | 31985078 |
| Other chemical reagents | Domestic, analytical grade |  |

## 2. Experimental Procedures

## 2.1 Cell Thawing and Culture

Culture media: HK2 cells, MEM supplemented with 10% fetal bovine serum (FBS) and 1% penicillin–streptomycin; THP-1 cells, RPMI 1640 supplemented with 10% FBS and 1% penicillin–streptomycin.

Remove the cells from liquid nitrogen and immediately place the cryovial in a 37 °C water bath. Gently shake the vial until the freezing medium has thawed.

Transfer the thawed cells to a centrifuge tube containing 10 mL of culture medium. Centrifuge at 1,200 rpm for 3 min at room temperature to collect the cells, then discard the supernatant.

Resuspend the cells in complete medium containing 10% FBS and 1% penicillin–streptomycin. Seed the cells in a culture dish and mix gently by pipetting. Incubate at 37 °C in 5% CO₂ under saturated humidity.

**2.2 Cell Passaging**

Passage the cells when they reach 80% confluence.

(1) Detach the cells with 0.25% trypsin. Stop trypsinization and collect the cells.

(2) Wash the cells twice with PBS, centrifuging at 1,200 rpm for 3 min.

(3) Add complete medium and pipette to obtain a single-cell suspension. Passage at a ratio of 1:3 and expand the cells at 37 °C in 5% CO₂ under saturated humidity.

**2.3 Transwell Migration Assay**

Cell seeding: Detach cells in the logarithmic growth phase and prepare a single-cell suspension in the appropriate culture medium. Seed the cells evenly into 6-well plates and incubate overnight at 37 °C in 5% CO₂ under saturated humidity.

Experimental groups:

HK2

HK2 + hyperuricemia

Sh-AR HK2 + hyperuricemia

Sh-NC HK2 + hyperuricemia

AR-OE HK2 + hyperuricemia

AR-NC HK2 + hyperuricemia

Preparation of a single-cell macrophage suspension: Detach the cells with trypsin and centrifuge at 1,500 rpm for 5 min at 4 °C. Discard the supernatant, resuspend the cells in serum-free medium, and count the cells.

Chamber preparation: Add 600 μL of complete medium to the lower chamber. Gently place the Transwell insert into the well containing the medium, taking care to avoid air bubbles.

Cell loading: Dilute the cell suspension with serum-free medium to 20,000 cells per 200 μL. Seed the cells from each group into the upper chamber of the Transwell insert.

Incubation: Incubate for a further 24 h at 37 °C in a 5% CO₂ incubator.

Cell staining: Discard the medium, add 600 μL of crystal violet staining solution per well, and stain for 15 min.

Washing: Carefully remove the cells from the upper chamber with a cotton swab. Rinse with tap water until no staining solution remains on the inner walls of the insert.

Imaging: Place the inserts upside down at a ventilation outlet and allow them to air-dry. Acquire microscopic images at 20× magnification.

3. Experimental Results

See the figure for details.
